# Suppression of poised oncogenes by ZMYND8 promotes chemo-sensitization

**DOI:** 10.1038/s41419-020-03129-x

**Published:** 2020-12-15

**Authors:** Shravanti Mukherjee, Santanu Adhikary, Shrikanth S. Gadad, Payel Mondal, Sabyasachi Sen, Ramesh Choudhari, Vipin Singh, Swagata Adhikari, Pratiti Mandal, Soumi Chaudhuri, Amrita Sengupta, Rajkumar Lakshmanaswamy, Partha Chakrabarti, Siddhartha Roy, Chandrima Das

**Affiliations:** 1 Biophysics and Structural Genomics Division, Saha Institute of Nuclear Physics, 1/AF Bidhannagar, Kolkata, 700064 India; 2 Structural Biology and Bioinformatics Division, CSIR-Indian Institute of Chemical Biology, 4 Raja S.C. Mullick Road, Kolkata, 700032 India; 3 Center of Emphasis in Cancer, Department of Molecular and Translational Medicine, Texas Tech University Health Sciences Center El Paso, El Paso, TX 79905 USA; 4 Graduate School of Biomedical Sciences, Texas Tech University Health Sciences Center El Paso, El Paso, TX 79905 USA; 5 Cecil H. and Ida Green Center for Reproductive Biology Sciences, Department of Obstetrics and Gynaecology, University of Texas Southwestern Medical Center, Dallas, TX 75390 USA; 6 Homi Bhaba National Institute, Mumbai, India; 7 Shri B. M. Patil Medical College, Hospital and Research Centre, BLDE (Deemed to be University), Vijayapura, Karnataka 586103 India; 8 Cell Biology and Physiology Division, CSIR-Indian Institute of Chemical Biology, 4 Raja S.C. Mullick Road, Kolkata, 700032 India

**Keywords:** Breast cancer, Gene silencing

## Abstract

The major challenge in chemotherapy lies in the gain of therapeutic resistance properties of cancer cells. The relatively small fraction of chemo-resistant cancer cells outgrows and are responsible for tumor relapse, with acquired invasiveness and stemness. We demonstrate that zinc-finger MYND type-8 (ZMYND8), a putative chromatin reader, suppresses stemness, drug resistance, and tumor-promoting genes, which are hallmarks of cancer. Reinstating ZMYND8 suppresses chemotherapeutic drug doxorubicin-induced tumorigenic potential (at a sublethal dose) and drug resistance, thereby resetting the transcriptional program of cells to the epithelial state. The ability of ZMYND8 to chemo-sensitize doxorubicin-treated metastatic breast cancer cells by downregulating tumor-associated genes was further confirmed by transcriptome analysis. Interestingly, we observed that ZMYND8 overexpression in doxorubicin-treated cells stimulated those involved in a good prognosis in breast cancer. Consistently, sensitizing the cancer cells with ZMYND8 followed by doxorubicin treatment led to tumor regression in vivo and revert back the phenotypes associated with drug resistance and stemness. Intriguingly, ZMYND8 modulates the bivalent or poised oncogenes through its association with KDM5C and EZH2, thereby chemo-sensitizing the cells to chemotherapy for better disease-free survival. Collectively, our findings indicate that poised chromatin is instrumental for the acquisition of chemo-resistance by cancer cells and propose ZMYND8 as a suitable epigenetic tool that can re-sensitize the chemo-refractory breast carcinoma.

## Introduction

Although a good initial response to chemotherapy is elicited in cancer patients, chemo-resistance still remains a major obstacle in successful cancer treatment. It is now well understood that chemotherapy kills a major fraction of tumor cells, but the drug-surviving cells “acquire” heightened chemo-resistance properties^1^. Therefore, understanding the mechanisms of gain in resistance properties in cancer cells post-chemotherapy could lead to improved outcomes of neoadjuvant chemo-therapies, thereby leading to enhanced disease-free survival. Recent reports have established that acquisition of chemo-resistance is also accompanied by a gain in stemness and tumor-promoting properties^2–4^. Moreover, chemo-evasive cells have been found to have a high expression of multidrug resistance (MDR) genes^5^. These studies have, therefore, raised concern among cancer therapists to address the issue of “acquisition of drug resistance” among tumor cell populations. In this context, it is very important to mention the contribution of cancer stem cells (CSCs). CSCs are a rare subpopulation of the tumor cells which have self-renewal and tumor-initiating potentials^6^ and are largely accountable for drug resistance and relapse in cancer^7,8^. Therefore, it is important to develop new strategies to circumvent chemo-resistance, by reverting the molecular phenotypes associated with it, thereby re-sensitizing the non-responsive breast cancer cells to chemotherapy.

Although chemotherapy is effective in eliminating breast cancer in pre-clinical studies, their clinical efficacy is mostly limited by toxic side effects^9^, and their efficiency is further constrained in highly invasive and therapy-resistant triple-negative breast cancer (TNBC) subtype. It is often found that patients are intolerant to the effective tumor elimination dose of the chemotherapeutic drug, while the levels that can be tolerated are therapeutically insufficient^10^. This shortcoming of chemotherapeutic treatment could be compensated by the use of specific therapeutic agents that will sensitize the cells to chemotherapy^11^, a phenomenon termed as chemo-sensitization. Aberrant epigenetic alterations have also been associated with the occurrence of tumorigenicity and drug resistance in cancer cells^12–16^. Quick activation or repression of genes, in response to external signals, is a characteristic feature of embryonic stem cells, which are endowed with “poised” chromatin states^17–19^. Poised promoters are characterized by the co-existence of both activating (H3K4Me3) and repressing (H3K27Me3) marks^19^. Limited studies have implied the existence of bivalent genes in cancer^20,21^. However, their implication in regulation of tumor biology is still poorly defined.

Epithelial to mesenchymal transition (EMT) is one of the key molecular mechanisms that promotes metastasis^22^. ZMYND8 (zinc finger, MYND domain-containing protein) is a putative chromatin reader, with tumor-suppressive functions^23,24^. Recent reports have suggested that through its reader function, ZMYND8 positively regulates epithelial gene expression^25^. It suppresses metastasis via its interaction with corepressors like KDM5C^26^. In the present study, we delineate the role of ZMYND8 as a potent repressor of chemo-resistance in breast cancer cells. A low dosage of chemotherapeutic drugs augment the tumorigenic potential of breast cancer cells. Overexpressing ZMYND8 transcriptionally represses the expression of drug resistance, stemness, and tumor-promoting genes by repressing their poised promoters in association with KDM5C and EZH2. Phenotypically it abrogates the breast CSC subpopulation, thereby leading to chemo-sensitization, both in vitro and in vivo. Therefore, our study establishes ZMYND8 as a potent chemo-sensitizer that can reverse chemo-resistance in breast cancer.

## Materials and methods

### Cell culture and chemotherapy treatment

MDA-MB-231, MDA-MB-468, HEK293T, and 4T1 cells were procured from the American Type Culture Collection (ATCC, USA). Cells were maintained in RPMI 1640 and Dulbecco’s modified Eagle’s medium (DMEM; Gibco, Invitrogen), respectively. All media were supplemented with 10% fetal bovine serum (Gibco) and 1% antibiotic–antimycotic (Gibco) at 37 °C in 5% CO_2_. For chemotherapy treatments, cells were treated with 0.6 µM doxorubicin or 10 µM 5-fluorouracil (5-FU) (Sigma) for 48 h in MDA-MB-231 cells and 1 µM doxorubicin or 20 µM 5-FU (Sigma) for 48 h in MDA-MB-468 cells.

### ZMYND8 overexpression and siRNA transfection

For ZMYND8 gene-silencing studies, RNA interference was carried out by using siRNA against ZMYND8 (smart-pool siRNA cocktail, Catalogue # sc-76337, Santa Cruz Biotechnology) or negative control siRNA (Santa Cruz Biotechnology) using INTERFERin transfection reagent (Polyplus) following the manufacturer’s protocol.

For overexpression of ZMYND8, 4 μg of FLAG-ZMYND8 (full length ZMYND8 cloned in pCMV-FLAG vector) was transfected per 10^5^ cells/well in a six-well plate using Lipofectamine 2000 (Invitrogen). After 24 h of transfection, the cells were harvested for subsequent analysis.

### ZMYND8 overexpression via lentiviral production

Full-length ZMYND8 was cloned into pCDH-CMVMCS-EF1-copGFP (CD511B-1) vector with *Eco*RI and *Bam*HI restriction sites. Recombinant lentivirus was produced as described previously^14^. Briefly, HEK293T cells were plated at 3 × 10^5^ density in 10-cm dishes and transfected with overexpression vectors along with packaging vector (psPAX2) and envelope vector (pMD2.G) using lipofectamine 2000 as per the manufacturer’s protocol. Post transfection, viral supernatant was harvested at 48 and 72 h. 4T1 cells were infected thrice in 48 h with the viral supernatant containing 10 μg/ml polybrene. Transduced cells were selected using Puromycin (4 μg/ml) (Sigma) for 3 days.

Doxycycline-inducible and lentiviral vectors for the inducible expression of ZMYND8 cDNA, or a GFP-cDNA control, were inserted into pInducer20^27^ and stably introduced into MDA-MB-231 cells under neomycin/G418 selection.

### Immunoblotting

Whole-cell lysates were prepared with Laemmli Buffer [4% SDS, 20% glycerol, and 20 mM Tris–HCl (pH 6.8)] and sonicated, followed by boiling at 100 °C for 5 min. The lysates were electrophoresed on 7.5%, SDS-PAGE gels. Blots were probed with specific antibodies. The membrane was blocked with 5% non-fat dry milk in TBST. Antibodies used are listed in Supplementary Table S1.

### Co-immunoprecipitation

Co-immunoprecipitation was performed, as described previously^23^. Briefly, the cells were lysed in lysis buffer, and immunoprecipitation was performed with specific antibodies. For DNase I co-immunoprecipitation, 500 µg of lysate was subjected to DNase I digestion at 37 °C for 1 h. The reaction was stopped by adding 5 mM EDTA. The DNA-free lysate was used for immunoprecipitation with specific antibodies. The immunoprecipitants were analyzed by immunoblotting.

### Sucrose-gradient protein fractionation

Sucrose-gradient fractionation was performed as described earlier^28^. Briefly, 10–40% sucrose gradients were formed by layering 400 μl of lysis buffer containing 10, 20, 30, or 40% sucrose in a sorvall ultracentrifuge tubes. One milligram of cell extract or 400 μg of molecular weight markers (Sigma MW-GF-1000) was loaded on top of the gradient and ultracentrifuged at 37,000 r.p.m. for 17 h at 4 °C using Sorvall WXUltra100 (Thermo Scientific) in an AH650 rotor. Forty fractions of 45 μl each were collected from the top, and alternate fractions were electrophoresed on 7.5% SDS-PAGE and analyzed by immunoblotting with desired antibodies.

### Chromatin immunoprecipitation (ChIP)

ChIP assays were performed as described earlier^23^. Cells were crosslinked with 1% formaldehyde, and the chromatin was sheared and immunoprecipitated with the desired antibodies or as a negative control IgG. ChIP DNA was analyzed by quantitative PCR (qPCR) using gene-specific primers. Each ChIP experiments were performed three independent times with technical triplicates. Primers used for ChIP assay are listed in Table S2.

### Quantitative real-time PCR (qRT-PCR)

Total RNA was isolated from cells by TRIzol (Invitrogen) and reverse transcribed by Revert aid Fast strand cDNA synthesis kit (Thermo Scientific) as per the manufacturer’s protocol. This was followed by qRT-PCR using ABI-SYBR GREEN mix (Applied Biosystems). qRT-PCR was performed using a StepONE plus FAST Real-time PCR machine. Each sample was analyzed three independent times and the results from one representative experiment, with technical triplicates, have been shown. Primers are listed in Supplementary Table S2.

### Cell migration assay

Cell migration assay was performed using 8.0-µm cell culture inserts (Thermo Scientific) as previously described^29^. Briefly, 2.5 × 10^5^ cells were seeded in serum-free media per well in the upper chamber of inserts, while the lower chamber contained complete media with 10% FBS. Cells were allowed to migrate for 8 h. Thereafter, the migrated cells were fixed and stained with giemsa, and image acquisition was done. Migrated cells were counted from three independent fields, and mean determined. The images were analyzed using the Image J software program (National Institutes of Health; NIH).

### Cell viability assay

Cell viability was measured by MTT assay. 5 × 10^4^ cells were seeded on a 12-well plate. After required treatments and transfections, fresh complete media were added to the plates, and 100 mg/ml MTT reagent was added to each well and incubated for 4 h. After that, the media was carefully discarded, and 150 ml of acidified isopropanol (4 mM HCl and 0.1% NP40 in isopropanol) was added to each well in order to dissolve the blue formazan crystals. The absorbance of this product was measured at 570 and 650 nm, using the ELISA plate reader (Stat Fax™® 2100 Microplate Reader, USA). The background reading at 650 nM was subtracted. As a blank, the cells received a 200 ml complete medium. The values of control sets were set as 100% cell viability and all the rest calculations were made relative to the control set.

### Mammosphere formation assay

Mammosphere formation assay was performed as described earlier^29^. Briefly, MDA-MB-231 cells were seeded in ultra-low attachment plates (Sigma Corning) at a density of 20,000 cells/ml, in serum-free DMEM-F/12 media supplemented with EGF, bFGF, insulin, BSA, and B27 (BD Biosciences). After 7 days, mammospheres were counted from three independent fields. The images were analyzed using the Image J software program (National Institutes of Health; NIH).

### Flow cytometry

Expression of human breast CSC markers CD44^+^/CD24^−^, ALDH1, and ESA in MDA-MB-231 cells and murine breast CSC markers CD44^+^/CD24^+^ in 4T1 cell-derived in vivo tumors were analyzed by flow cytometry as described previously^29^.

### In vivo tumorigenicity and chemo-sensitization assays

Six to eight weeks old female Balb/c were housed in individually ventilated cages under alternate dark and light cycles and maintained on food and water in the central animal house facility of CSIR-Indian Institute of Chemical Biology (CSIR-IICB). Mice were anesthetized by injecting Averdin solution intraperitoneally at a volume of 400 μL per 18–20 g of animal weight. The fur was shaved over the flanks, and 1 × 10^5^ 4T1 cells suspended in 0.2-ml PBS were injected (subcutaneously) into both sides of the flank^30^. After 7–10 days, when primary tumors were visible, 100 μL of doxorubicin (8 mg/kg body weight) was injected intraperitoneally on alternative days. After three doses of doxorubicin injection, mice were sacrificed; tumors were excised and measured by slide calipers. Tumor volumes were calculated using the formula *π*/6 ((*d*1 × *d*2)3/2), where *d*1 and *d*2 are the two perpendicular diameters^29^. Single-cell preparation from excised tumors for flow cytometric analysis and RNA isolation was performed as described earlier^29^. All animals were treated in accordance with the guidelines of the Institutional Animal Ethics Committee (approved by CPCSEA, Govt. of India) of CSIR-IICB. For each group, five mice were used for statistical significance.

### Xenograft experiments

Animal experiments were performed in compliance with the Institutional Animal Care and Use Committee (IACUC) at the Texas Tech University Health Sciences Center El Paso. Female nude mice (Jackson laboratories: 002019-NU/J) at 4–6 weeks of age were used. We used female mice because mammary cancers occur primarily in females. In addition, the human cancer cell lines that we used for xenografts are from females. For xenograft experiments, a similar protocol was followed as in the case of our 4T1 murine tumor model, except that two cycles of chemotherapy were administered. Mouse weight was monitored, and tumor growth measured over time using electronic calipers approximately every 2–3 days. Tumor volumes were estimated by the following formula: tumor volume = (width^2^ × length)/2. Animals were euthanized after seven days post-two cycles of chemotherapy.

### RNA-sequencing

#### Sample preparation

For library construction, total RNA was extracted from the samples by RNeasy kit. After initial quality control, the library was constituted. The extracted RNA with an RNA integrity number of ≥7.0 was used for mRNA purification. The mRNA was purified using oligo-dT beads (TruSeq RNA Sample Preparation Kit, Illumina) from 1 μg of intact total RNA. The purified mRNA was fragmented at 90 °C in the presence of divalent cations. The fragments were reverse transcribed using random hexamers and Superscript II Reverse Transcriptase (Life Technologies). Second-strand cDNA was synthesized on the first-strand template using RNaseH and DNA polymerase I. The sequencing library was prepared by 5′ and 3′ adapter ligation, after end-repair and the addition of an “A” base and SPRI clean up. The prepared cDNA library was amplified using PCR for the enrichment of the adapter-ligated fragments. The individual libraries were quantified using a Qubit fluorometer and validated for quality with a Bioanalyzer (Agilent Technologies). SPRI-based (Beckman Coulter Agencourt Ampure XP) purification was used to clean after each enzymatic reaction.

#### RNA-Seq data analysis

Post sequencing analyses were performed by Kinsight Bio Analytics LLC. Following initial quality checks, the reads were aligned with the UCSC Human genome (hg38) reference genome using TopHat pipeline^31^ with a Bowtie2 index. The treated sample was compared with that of the control sample using cuffdiff^32^. Transcripts with log2 fold change cutoff of 1 and *P* value ≤ 0.05 were considered as significantly differentially expressed. Gene ontologies and pathways that harbor significantly expressed transcripts were identified using DAVID Functional Annotation Tool.

Gene ontologies and pathways that harbor significantly regulated transcripts were also identified using GSEA (gene set enrichment analysis) tool. (http://software.broadinstitute.org/gsea/msigdb/annotate.jsp).

### Accession numbers

The accession number for the RNA-seq datasets generated for this study is NCBI-GEO: GSE145141.

### Kaplan–Meier and gene expression analyses in patient tumor samples

Kaplan–Meier plots were generated using the Gene Expression-Based Outcome for Breast Cancer Online (GOBO) tool (http://co.bmc.lu.se/gobo/)^33^. Gene expression levels in patient tumor samples were also assessed using the GOBO tool.

### Bioinformatics analyses

For the analysis of clinical data, previously published datasets were obtained from ArrayExpress. Microarray data from pre-neoadjuvant trial of cisplatin monotherapy in TNBC tumors were obtained from GEO ID: GSE18864^34^. Datasets of reference tumors (primary breast tumor samples, not having undergone trial) were compared to that of patients showing either no or minimal reduction in the tumor (Miller-Payne scale 1) or significant (either >90% or complete remission) reduction in tumor size (Miller-Payne scale 4 and 5). Sample IDs and corresponding expression values have been provided under Supplementary Table S3. In another clinical analysis, the comparison was made between seven TNBC patients with tumor recurrence after neoadjuvant chemotherapy versus 7 TNBC patients, with no tumor recurrence after similar chemotherapy regime (GSE43502)^35^. Sample IDs and corresponding expression values have been provided under Supplementary Table S4. GEO2R function of the NCBI-GEO database was used for identifying differentially expressed genes of the clinical datasets. ChIP-Seq data for ZMYND8 (RACK7) were obtained from publicly available datasets in ArrayExpress (GSE71323), performed in ZR-75–30 cells^26^. List of promoter bivalent chromatin modifications in human embryonic stem cells (hESCs) was obtained from previously published works of Court et al.^36^. The authors have collected and combined publicly available data set (sourced from NIH Roadmap Epigenomics project) for H3K27Me3 and H3K4Me3 from five different hESC cell lines (HUES48, HEUS64, HEUS6, I3, and H1) in order to generate high confidence bivalent chromatin domains in hESC genome.

### Statistical analyses

All data have been expressed as mean ± standard deviation (s.d.), and the s.d. are represented by error bars. The statistical significance was calculated by either unpaired Student’s *t*-test, one-way Anova, or two-way Anova as specifically mentioned. *P* value ≤ 0.05 was considered as significant. The experiments were done at least three times in duplicate unless otherwise stated.

## Results

### Loss of ZMYND8 promotes stemness, drug resistance, and EMT

**Fig. 1:**
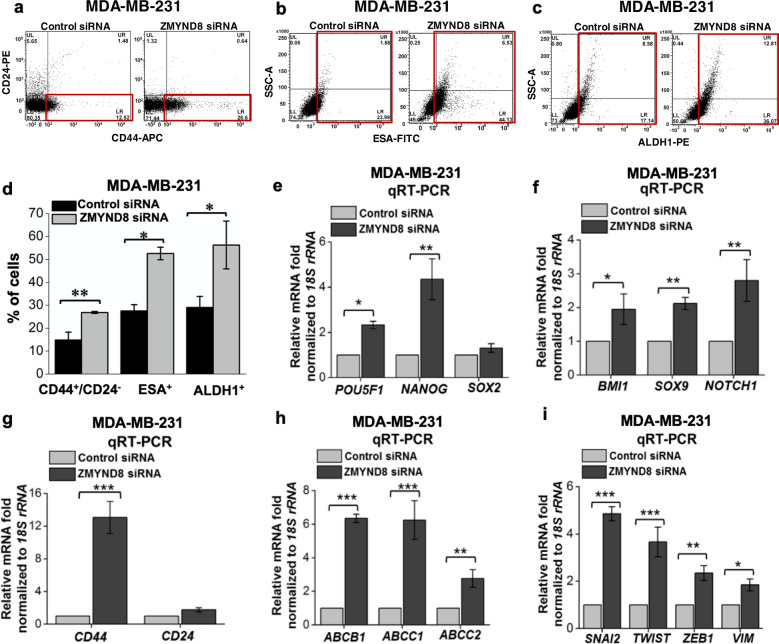
ZMYND8 suppresses EMT, drug resistance, and stemness. **a–d** FACS analysis showing bCSC (CD44^+^/CD24^−^) (**a**), ESA^+^ (**b**), and ALDH1^+^ (**c**) cells upon knockdown of ZMYND8 in MDA-MB-231 cells. The percent of cells have been quantified and represented graphically (**d**). **e–i** qRT-PCR analysis showing the expression of pluripotency/stemness related (**e**–**g**), drug resistance (**h**), and EMT (**i**) genes upon ZMYND8 knockdown via siRNA in MDA-MB-231 cells. In panels, **d–i** error bars indicate standard deviation (s.d.); *n* = 3, a representative with technical replicates (out of three experiments). *P* values were calculated using unpaired Student’s *t*-tests. *P < 0.05; **P < 0.01; ***P < 0.001.

Recent evidences suggest that EMT triggers the poorly differentiated cancer cells to gain stemness and MDR properties^37–39^. ZMYND8 has been previously reported to be a potent tumor suppressor by inhibiting proliferation and metastasis^24–26,30^. Therefore, we wanted to investigate the anti-cancer role of ZMYND8 in the context of drug resistance and the stemness properties of tumor cells. Genetic depletion of *ZMYND8* in MDA-MB-231 cells led to an increase in breast CSC (bCSC) (CD44^+^/CD24^−^) content^40^ as well as expression of other stemness markers, ESA and ALDH1^40,41^ as determined by FACS analysis (Fig. 1a–d). Similarly, we found upregulation in the expression of pluripotency (*POUSF1*, *NANOG*, *SOX2*, *BMI1*, *SOX9*, and *NOTCH1*), drug resistance (*ABCB1*, *ABCC1*, and *ABCC2*), EMT (*SNAI2*, *TWIST1*, *ZEB1*, and *VIM*), and stemness (*CD44* and *CD24*)-related genes upon knockdown of ZMYND8 by smart-pool siRNA in invasive breast cancer cells MDA-MB-231 and MDA-MB-468 (Fig. 1e–i and Supplementary Fig. 1). Thus, our results indicate the potential role of ZMYND8 in suppressing gain in stemness and drug resistance properties of tumor cells, a phenomenon that is molecularly governed by EMT.

### ZMYND8 negatively regulates low-dose chemotherapy-dependent migration and stemness of tumor cells by altering tumor-promoting gene expression profile

**Fig. 2:**
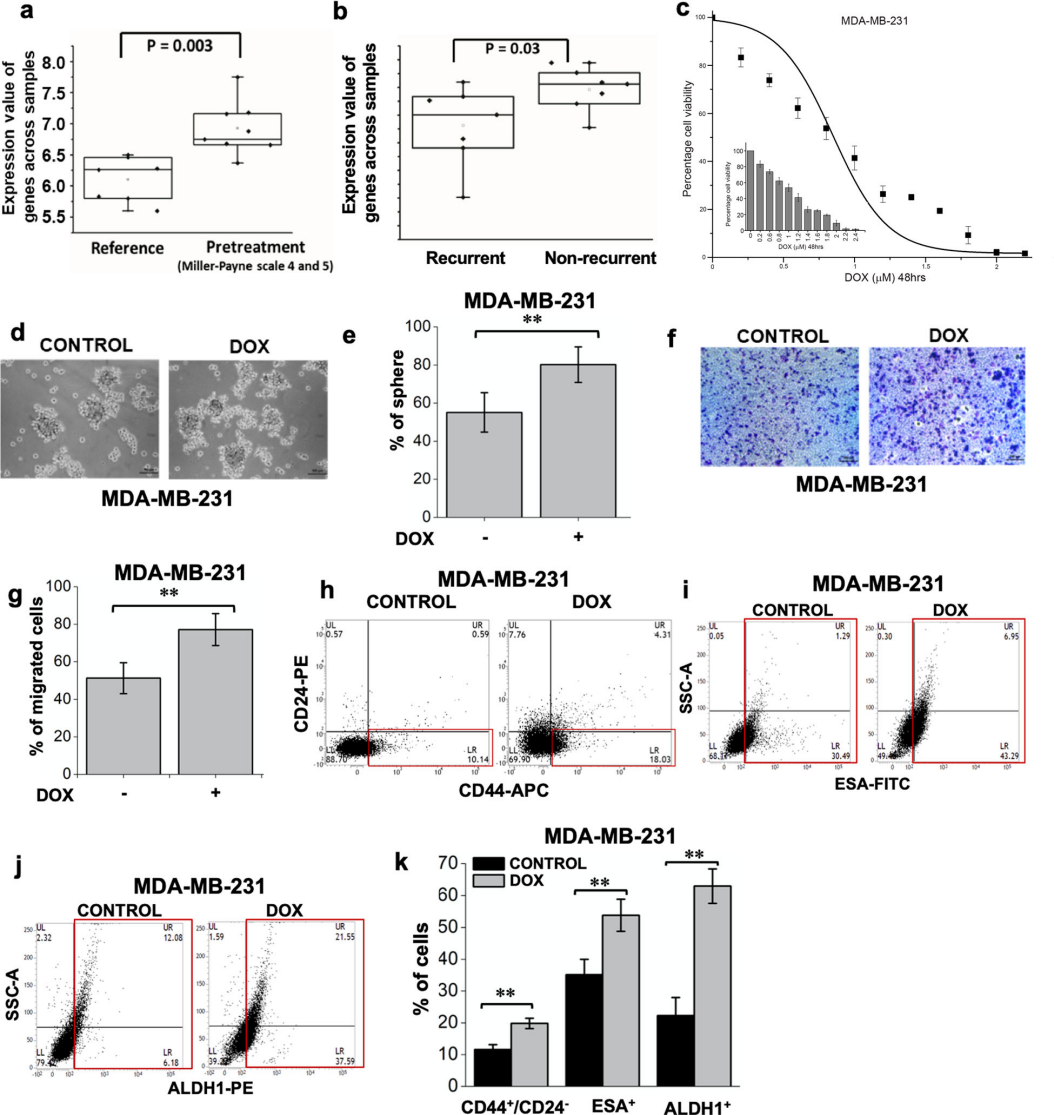
Low-dose chemotherapy induces EMT, drug resistance, and stemness. **a**, **b** Box plot of ZMYND8 expression in primary breast tumor (no clinical trial) compared to pre-treatment TNBC patient tumor samples having a good pathological response (Miller–Payne score 4 and 5) (**a**). Box plot of ZMYND8 expression from tumor samples of TNBC patients having a recurrence of tumor post-neoadjuvant chemotherapy, compared to TNBC patients having no recurrence after a similar regime of chemotherapy (**b**). **c** MDA-MB-231 cells were treated with increasing doses of doxorubicin for 48 h, and cell viability was calculated by MTT assay. **d**–**g** Mammosphere formation (**d**, **e**) and Transwell migration assay (**f**, **g**) was carried out with MDA-MB-231 cells treated with 0.6 µM doxorubicin for 48 h. Scale bar represents 100 µm. **h**–**k** FACS analysis showing bCSC (CD44^+^/CD24^−^) (**h**), ESA^+^ (**i**), and ALDH1^+^ (**j**) cells upon 0.6 µM doxorubicin treatment for 48 h in MDA-MB-231 cells. In panels **e**, **g** and **k** error bars indicate standard deviation (s.d.); *n* = 3, a representative with technical replicates (out of three experiments). *P* values were calculated using unpaired Student’s *t*-tests. *P < 0.05; **P < 0.01; ***P < 0.001.

Previous studies have shown that ZMYND8 levels are significantly lower in aggressive breast cancers, such as basal subtype tumors and higher levels in luminal breast cancers^42^. However, ZMYND8 expression was found to be significantly elevated in TNBC patients with a good pathological response (Miller–Payne score 4 and 5) as compared to reference patient cohort^34^ (Fig. 2a). Furthermore, post neoadjuvant chemotherapy treatment, ZMYND8 expression was higher in non-relapsed TNBC patients, compared to relapsed (Fig. 2b), indicating that ZMYND8 expression is higher in chemo-sensitive tumors compared to chemo-resistant tumors. These observations prompted us to investigate ZMYND8’s role in chemotherapy treatment of cancer cells from a molecular perspective. We used two well-established chemotherapeutic drugs, doxorubicin and 5-FU, and performed dose-dependent survival assay and determined the IC_50_. The sublethal concentration of 0.6 and 1.0 µM of doxorubicin in MDA-MB-231 (IC_50_: 0.754 µM) and MDA-MB-468 (IC_50_: 1.69 µM) cells respectively were chosen for subsequent assays (Fig. 2c and Supplementary Fig. 2a). Similarly, 10 and 20 µM of 5-FU in MDA-MB-231 (IC_50_: 15.53 µM) and MDA-MB-468 (IC_50_: 42.62 µM) cells, respectively, were used (Supplementary Fig. 2a, c). To understand whether the drug-resistant phenotypes in MDA-MB-231 cells were elicited by a sublethal dose of doxorubicin, we performed in vitro mammosphere formation assay, which showed an enhanced spheroid formation ability compared to untreated cells (Fig. 2d, e). Similarly, a sublethal dose of doxorubicin treatment increased the migration as well as the population of CD44^+^/CD24^−^, ESA^+^, or ALDH1^+^ MDA-MB-231 cells (Fig. 2f–k). Also, sublethal doses significantly increased the expression of tumor-promoting genes, drug resistance, and stemness post 48 h treatment (Supplementary Fig. 2d–g). These findings indicated that doxorubicin treatment led to the enrichment of the CSC pool of the tumor, which are the key players in drug resistance and tumorigenicity.

### ZMYND8 regulates doxorubicin-dependent gene expression profile to effect chemo-sensitization

**Fig. 3:**
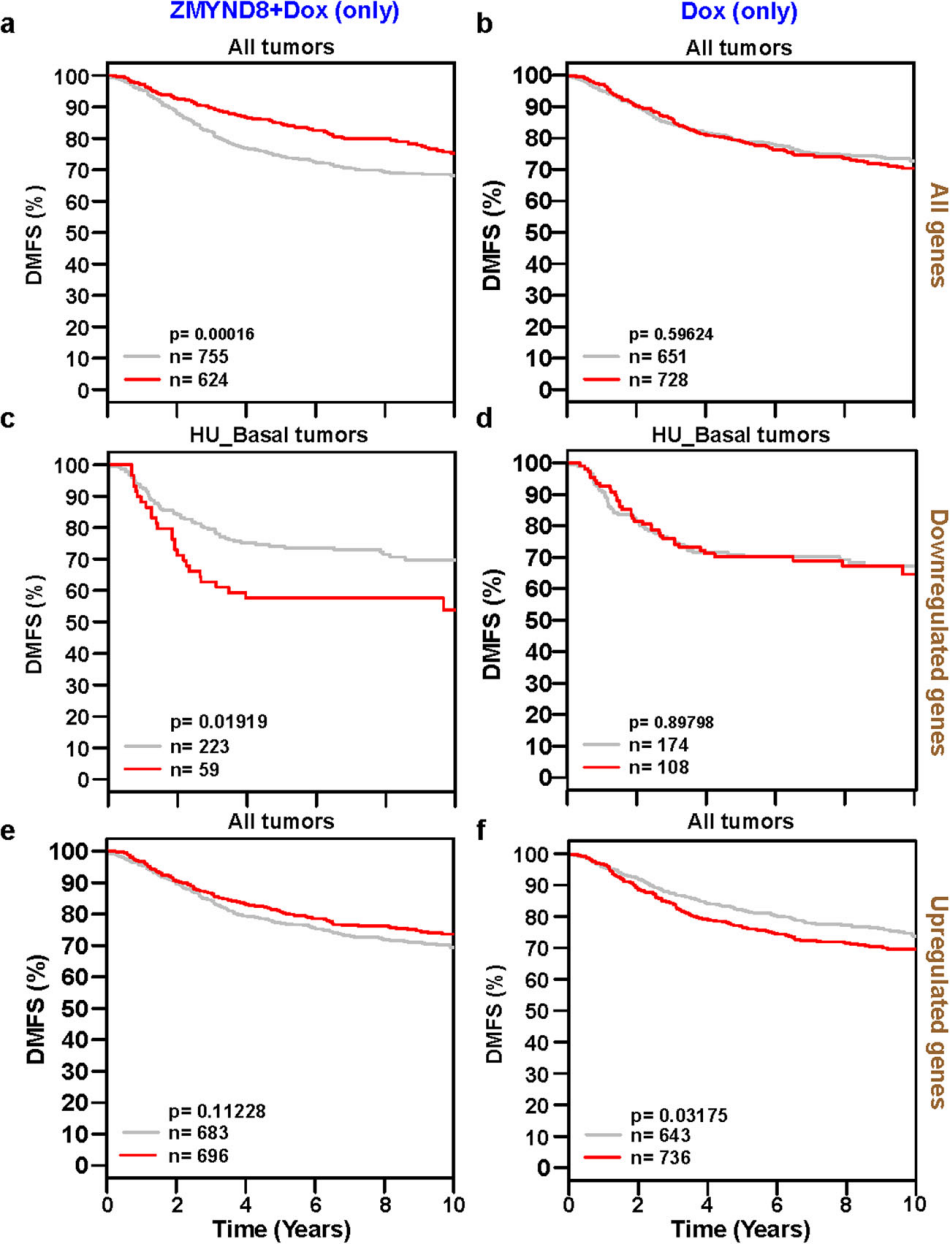
ZMYND8/doxorubicin-specific mRNA expression profile is predictive of clinical outcomes in breast tumor patients. **a** Kaplan–Meier survival analyses of patients expressing high levels of ZMYND8/doxorubicin-specific regulated *mRNAs* (red line) exhibit a better distant metastasis-free survival (DMFS) compared to patients expressing low levels of coregulated gene *mRNAs* (gray line). **b**, **d**, **e** The expression of ZMYND8/doxorubicin- or doxorubicin-specific genes exhibit no effect on outcome in breast cancer patients. **c** Kaplan–Meier survival analyses of patients expressing high levels of ZMYND8/doxorubicin-specific downregulated *mRNAs* (red line) exhibit a poorer distant metastasis-free survival (DMFS) compared to patients expressing low levels of downregulated gene *mRNAs* (gray line). **f** High levels of doxorubicin-specific upregulated *mRNAs* (red line) exhibit a lower DMFS compared to patients expressing low levels of upregulated *mRNAs* (gray line). The breast cancer outcome-linked gene expression data were accessed and graphed using the Gene Expression-Based Outcome for Breast Cancer Online (GOBO) tool^33^.

Since we observed that a lower dose of chemotherapy led to gain in chemo-resistance, migration, and stemness, we hypothesized that ectopic expression of ZMYND8 could lead to the reversal of tumor-promoting phenotypes. We also observed that ZMYND8 expression was low in the basal subtype of breast cancer cells (MDA-MB-231 and MDA-MB-468) when compared to MCF-7 cells (Supplementary Fig. 3a). In order to determine whether ZMYND8 can chemo-sensitize MDA-MB-231 cells by regulating tumor-related genes, we performed genome-wide, transcriptome analysis with or without doxorubicin treatment in MDA-MB-231 cells upon transiently transfecting ZMYND8 expression vector. We found that 967 genes (coding and noncoding) were differentially regulated upon doxorubicin treatment in the absence or presence of ZMYND8, as assessed by Venn diagram, volcano plot, and heatmap analyses (Supplementary Fig. 3b, c). Of 967 genes, 568 were regulated by both doxorubicin treatment alone or in combination with ZMYND8 overexpression in MDA-MB-231 cells (Supplementary Fig. 3b). However, the magnitude of gene regulation was significantly higher in doxorubicin-treated ZMYND8-overexpressed MDA-MB-231 cells when compared to doxorubicin alone (Supplementary Fig. 3d), which suggests that ZMYND8 has an additive effect on the doxorubicin-regulated transcriptome. We subsequently performed gene ontology (GO) analysis on the 568 genes (having *P* value ≤0.05, log2 fold change ≥1) (Supplementary Figs. 3e and 4). GO analysis of these 568 genes suggested their role in cancer, cell cycle, p53, and Akt-signaling (Supplementary Fig. 3e), and they are upregulated in basal subtype (Supplementary Fig. 3f) and also in higher grade breast tumors (Supplementary Fig. 3g). Additionally, Kaplan–Meier analysis of these genes indicated poor prognosis in breast cancer patients (Supplementary Fig. 3h). Further, we elucidated the role of ZMYND8 in doxorubicin-dependent gene regulation and its association with patient outcomes. Intriguingly, the subset of genes that were regulated upon ZMYND8 overexpression in combination with doxorubicin treatment showed higher distant metastasis-free survival (DMFS) (Fig. 3a), as assessed by Kaplan–Meier analysis in breast cancer patients. However, doxorubicin-specific regulated genes alone had no effect on DMFS (Fig. 3b). Moreover, ZMYND8 overexpression in doxorubicin-treated cells attenuated the expression of genes (top downregulated) associated with poor prognosis in basal subtype breast cancer patients (Fig. 3c) compared to doxorubicin-downregulated genes (Fig. 3d). On the contrary, ZMYND8 and doxorubicin upregulated genes had minimal effect on prognosis in breast cancer patients (Fig. 3e) compared to doxorubicin-upregulated genes (Fig. 3f**)**. These analyses indicate that ZMYND8 overexpression in MDA-MB-231 cells treated with doxorubicin significantly alters the tumor/cancer-related gene levels and signaling pathways specific to aggressive breast cancer, and suggests the role of ZMYND8 in chemo-resistance.

**Fig. 4:**
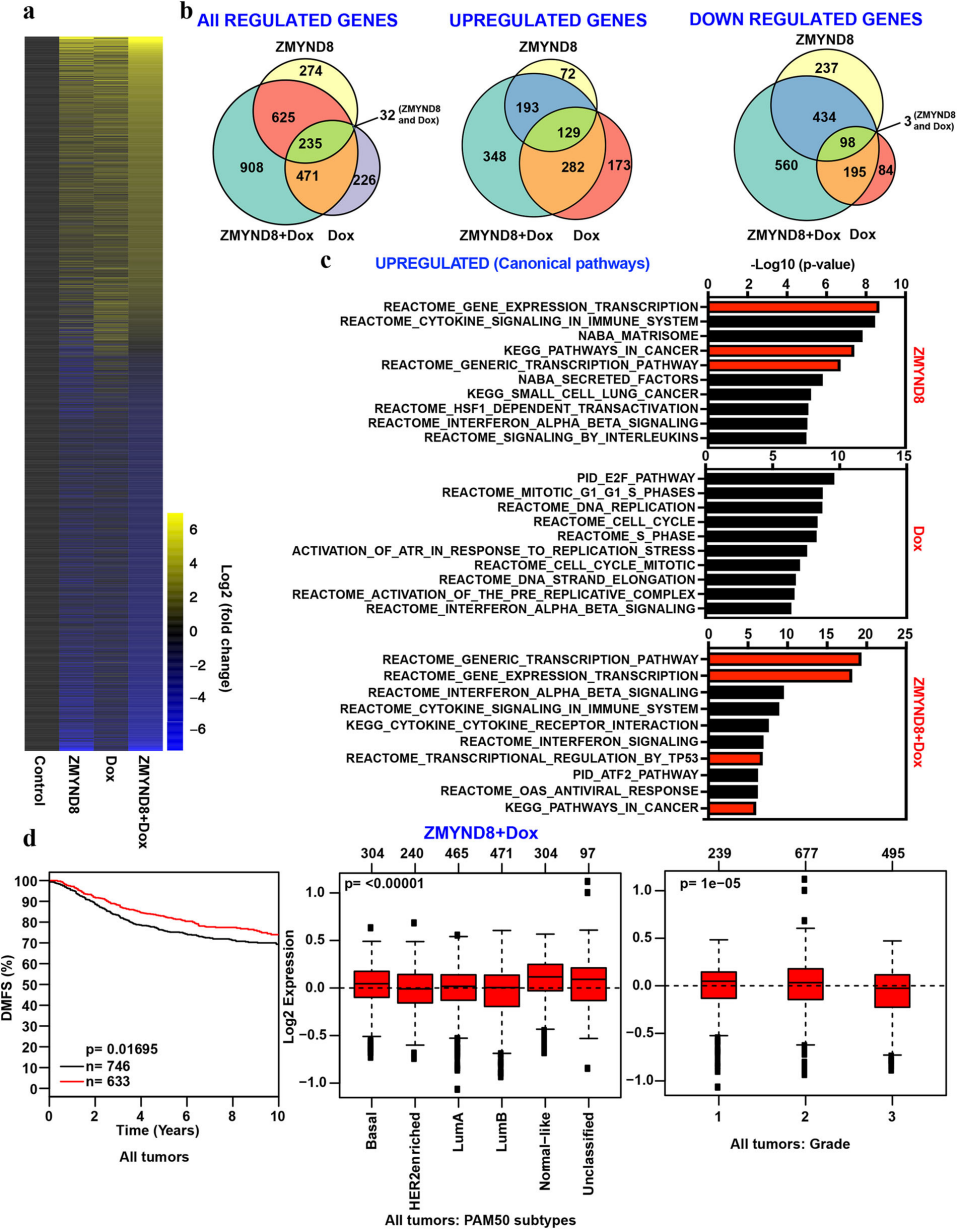
Genome-wide gene expression changes upon ZMYND8 overexpression and/or doxorubicin treatment. **a** Heatmap of differentially expressed genes identified through RNA-seq analysis (log2 fold change ≥1.5, and *P* value ≤ 0.05) upon transient ZMYND8 overexpression and/or doxorubicin treatment (0.6 µM for 48 h) in MDA-MB-231 cells using a doxycycline-inducible expression system. **b** Venn diagram depicting the number significantly all-, up-, and downregulated genes (log2 fold change ≥1.5, and *P* value ≤0.05). **c** GSEA (gene set enrichment analysis) for canonical pathways showing the highest enrichment in a list of DEGs obtained after RNA-seq analysis of MDA-MB-231 cells upon different treatment conditions. Canonical pathways are indicated on the *Y*-axis. *P* value at *X*-axis indicates the significance level of each pathway as obtained using http://software.broadinstitute.org/gsea/msigdb/annotate.jsp. **d** Kaplan–Meier survival analyses of patients expressing high levels of ZMYND8/doxorubicin-upregulated gene mRNAs (red line) exhibit a good outcome compared to patients expressing low levels of upregulated gene mRNAs (black line). Box plots showing the ZMYND8/doxorubicin-upregulated genes expressed in patient tumor samples of normal-like or lower-grade breast cancer subtype. Observed differences are significant as determined by an ANOVA comparison of the means (*P* value < 0.00001 or *P* value = 1e−05). The breast cancer outcome-linked gene expression data were accessed and graphed using the Gene Expression-Based Outcome for Breast Cancer Online (GOBO) tool^63^.

Furthermore, we sought to identify the independent roles of ZMYND8 and doxorubicin as well as their combined effect in defining the gene expression patterns in MDA-MB-231 cells. For this, we created inducible ZMYND8 or GFP overexpressing MDA-MB-231 cell lines (Supplementary Fig. 5a, b). Unlike the cells that were transfected with ZMYND8 plasmid, the inducible cell lines with transiently elevated ZMYND8 levels without the side effects of transfection helped us to determine its independent role more clearly in chemo-resistance. Using these cell lines, we performed RNA-seq analysis, and the results showed that 1166 and 965 genes were differentially regulated by ZMYND8 or doxorubicin alone, respectively (Fig. 4a, b). However, doxorubicin, in combination with ZMYND8 overexpression, affected 2239 candidate genes (Fig. 4b), the majority of them were up- or down-regulated (Fig. 4b). Further, GSEA (Gene Set Enrichment Analysis) analyses of ZMYND8-positively-regulated genes suggest that ZMYND8 alone or in combination with doxorubicin positively regulates gene transcription and cancer pathways (Fig. 4c and Supplementary Fig. 5c), whereas doxorubicin alone affected cell-cycle-related pathways (Fig. 4c) indicating its role in replication stress and DNA damage. Of note, we found that similar to previously published findings, ZMYND8 alone regulates the expression of candidate DNA-damage-related genes such as *BRCA1*, *CDC25A*, *MCM10*, *RFC3*, and *CLSPN* (Supplementary Fig. 5d). However, ZMYND8 overexpression had a minimal or insignificant additive effect on their expression in the presence of doxorubicin (Supplementary Fig. 5d). Further, Kaplan–Meier analysis showed that ZMYND8 independently or in the presence of low dose of doxorubicin, induced gene expression profiles that are associated with better DMFS (Fig. 4d and Supplementary Fig. 6c), and these genes are preferentially expressed in normal-like or low grades of breast tumors (Fig. 4d and Supplementary Fig. 6d). However, the doxorubicin-upregulated genes had no effect on clinical outcome (Supplementary Fig. 6c), and they were preferentially expressed in the basal subtype of breast tumors (Supplementary Fig. 6d). Additional analyses of the top 20 negatively regulated genes in all the conditions are preferentially expressed in less aggressive breast cancer subtypes. However, their expression had no association with clinical outcomes (Supplementary Fig. 6a, b). Collectively, the gene expression analyses using both the systems of ZMYND8 overexpression suggest its positive role in combating low-dose doxorubicin-induced chemo-resistance.

### Chemotherapeutic treatment in the presence of ZMYND8 overexpression significantly abrogates tumor-promoting phenotypes

**Fig. 5:**
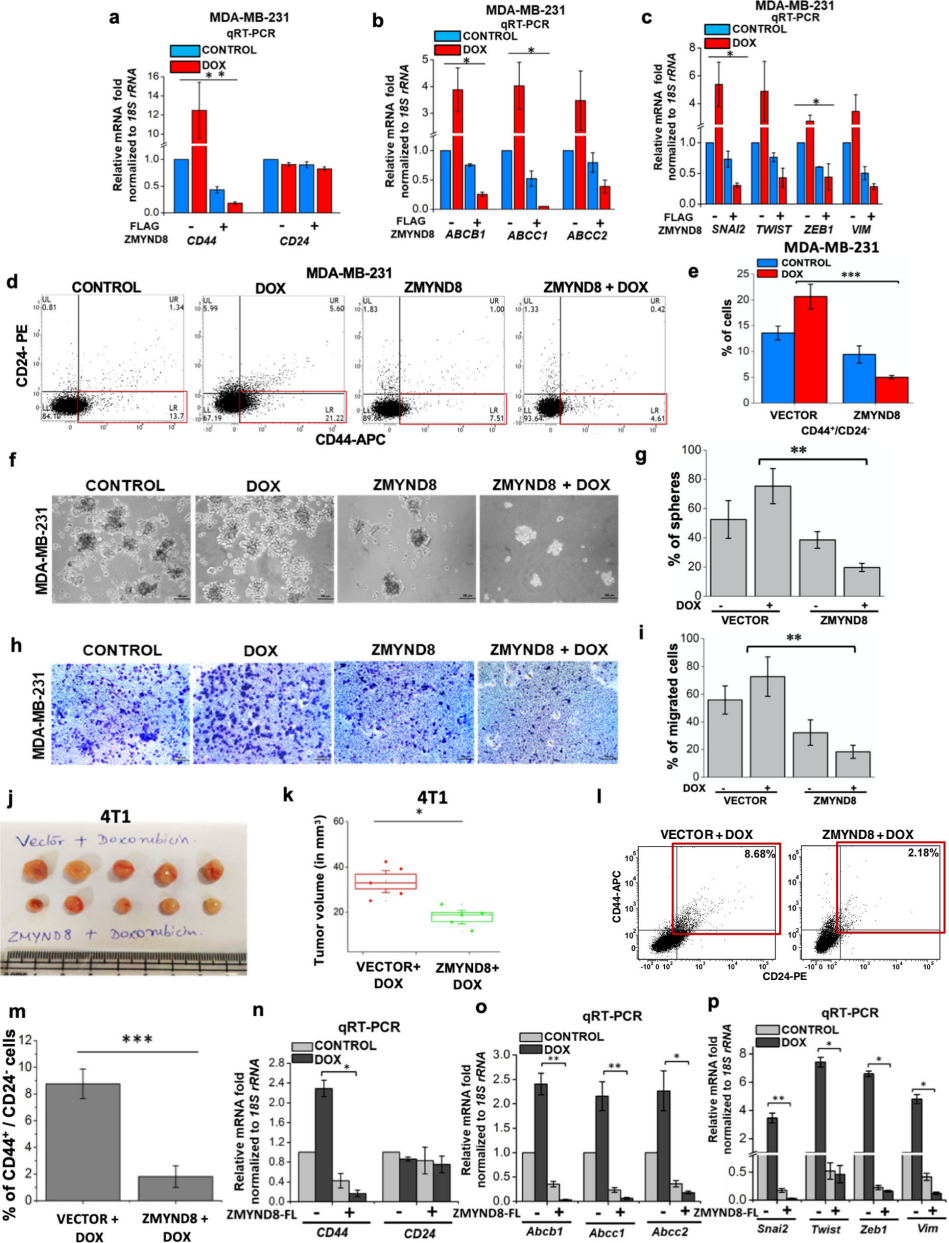
ZMYND8 promotes the chemo-sensitization of triple-negative breast cancer towards chemotherapy. **a–c** qRT-PCR analysis showing expression of stemness (**a**), drug resistance (**b**), and EMT (**c**) genes upon ectopic expression of ZMYND8 followed by 0.6 µM doxorubicin treatment for 48 h in MDA-MB-231 cells. **d**–**i** FACS analysis showing bCSC (CD44^+^/CD24^−^) cells (**d**, **e**), mammosphere formation (**f**, **g**), and trans-well migration assay (**h**, **i**) from ZMYND8 overexpressed followed by 0.6 µM doxorubicin treatment of MDA-MB-231 cells for 48 h. Scale bar represents 100 µm. **j**, **k** In vivo tumor formation assay with 4T1 cells expressing either vector or ZMYND8. Post tumor development, mice were administered with doxorubicin at a dose of 8 mg/kg body weight every alternate day three times. Representative image of the tumors 7 days of post-three cycles of chemotherapy (**j**). Graphical representation of tumor growth rate (**k**). *n* = 4 mice per group. **l**–**m** FACS analysis showing bCSC (CD44^+^/CD24) cells from mouse tumor. **n**–**p** qRT-PCR analysis showing the expression of stemness (**n**), drug resistance (**o**) and EMT (**p**) related genes from mouse tumors. In panels **a–c**, **e**, **g**, **i**, **m**–**p** error bars indicate standard deviation (s.d.); *n* = 3, a representative with technical replicates (out of three experiments). *P* values were calculated using one-way ANOVA. *P < 0.05 and **P < 0.01.

Genome-wide transcriptome analysis indicated that ectopic expression of ZMYND8 can prolong the DMFS by altering the global gene expression programs triggered by doxorubicin. We next correlated the changes in the gene expression levels of drug resistance, EMT, and stemness-promoting genes with the observed chemo-sensitization phenomenon in both MDA-MB-231 and MDA-MB-468 cells. Ectopic expression of ZMYND8 followed by doxorubicin or 5-FU treatment led to downregulation of genes involved in stemness, drug resistance, and EMT (Fig. 5a–c and Supplementary Fig. 7a–i). Immunoblot analysis of EMT marker under similar conditions further confirmed the restoration of the epithelial state of the cell (Supplementary Fig. 7j). We next evaluated whether gene-transcription changes initiated by ZMYND8 overexpression in doxorubicin-treated cells are also reflected in tumor regression phenotypes. Series of gain-of-function experiments showed that ectopic expression of ZMYND8 followed by doxorubicin treatment in MDA-MB-231 cells led to significant regression in bCSC (CD44^+^/CD24^−^) content along with a decrease in expression of other stemness markers, ALDH1 and ESA (Fig. 5d–e and Supplementary Fig. 8a–d), consequently manifesting a compromised mammosphere formation (Fig. 5f, g) and migratory potential (Fig. 5h, i) of the cancer cells. We next validated our findings in vivo both in syngeneic and xenograft tumor models and observed that ZMYND8-overexpressed tumors significantly regressed compared to control (empty vector expressed) tumors, upon doxorubicin treatment in both tumors derived from ZMYND8 overexpressing 4T1 (Fig. 5j, k) and MDA-MB-231 (Supplementary Fig. 8e–g) cells. Furthermore, a decrease in murine bCSC content (CD44^+^/CD24^+^) was observed in doxorubicin-treated ZMYND8-overexpressed tumors as compared to doxorubicin alone (Fig. 5l, m). Similar to in vitro analyses, ex vivo analyses of the tumors showed that ZMYND8 overexpression led to a significant downregulation of MDR, EMT, and stemness genes (Fig. 5n–p). Cumulatively, in vitro and in vivo results prove that tumor suppressor ZMYND8 chemo-sensitizes via transcriptional repression of drug resistance, migration, and stemness genes.

### ZMYND8 chemo-sensitizes by altering poised epigenetic state to repressed state, aided by corepressors KDM5C and EZH2

**Fig. 6:**
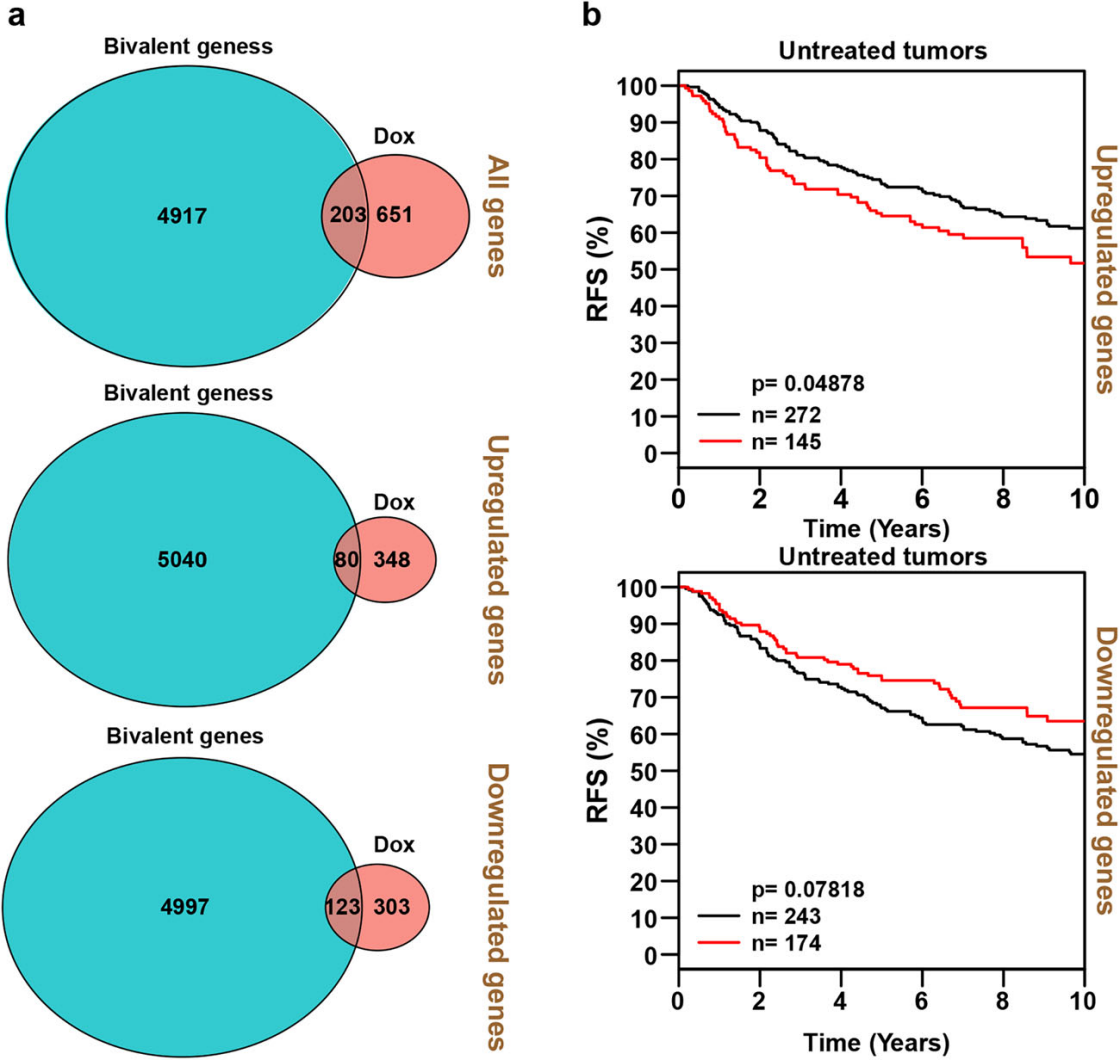
Activation of “poised” tumor-promoting genes underlies doxorubicin-induced rapid gain in resistance, stemness, and migration phenotypes. **a** Venn diagram depicting common genes with poised or bivalent promoters that are regulated upon doxorubicin treatment in MDA-MB-231 cells. **b** Kaplan–Meier survival analyses of patients expressing high levels of doxorubicin-upregulated *bivalent gene mRNAs* (red line) exhibit a poorer relapse-free survival (RFS) compared to patients expressing low levels of *coregulated gene mRNAs* (black line) (top panel). High levels of doxorubicin-downregulated *mRNAs* (red line) tend to exhibit a better RFS compared to patients expressing low levels of *coregulated gene mRNAs* (black line). The breast cancer outcome-linked gene expression data were accessed and graphed using the Gene Expression-Based Outcome for Breast Cancer Online (GOBO) tool^33^.

The prompt phenotypic as well as global transcriptional changes observed in ZMYND8 overexpressed cells subjected to a sublethal dose of doxorubicin led us to propose that a malleable chromatin landscape could be instrumental in modulating such changes. In hESCs, such transcriptional regulation is mediated by poised epigenetic states at gene promoters^19^. Although bivalent genes have been reported in cancer cells^20,21^, however, their regulatory mechanism and detailed categorization in cancer cells has not been studied in tumor biology. Interestingly, when we compared the total number of bivalent promoter containing genes in MDA-MB-231 cells with the doxorubicin-regulated gene set from our transcriptomic analysis, we identified substantial overlap (Fig. 6a). Subsequent Kaplan–Meier analysis suggests that doxorubicin-upregulated bivalent genes are associated with significantly lower relapse-free survival (RFS) in untreated breast cancer patients (Fig. 6b), whereas doxorubicin-downregulated bivalent genes have an opposite trend on RFS in breast cancer patients (Fig. 6b). Taken together, our analysis indicates that doxorubicin induces poised tumor-promoting genes.

**Fig. 7:**
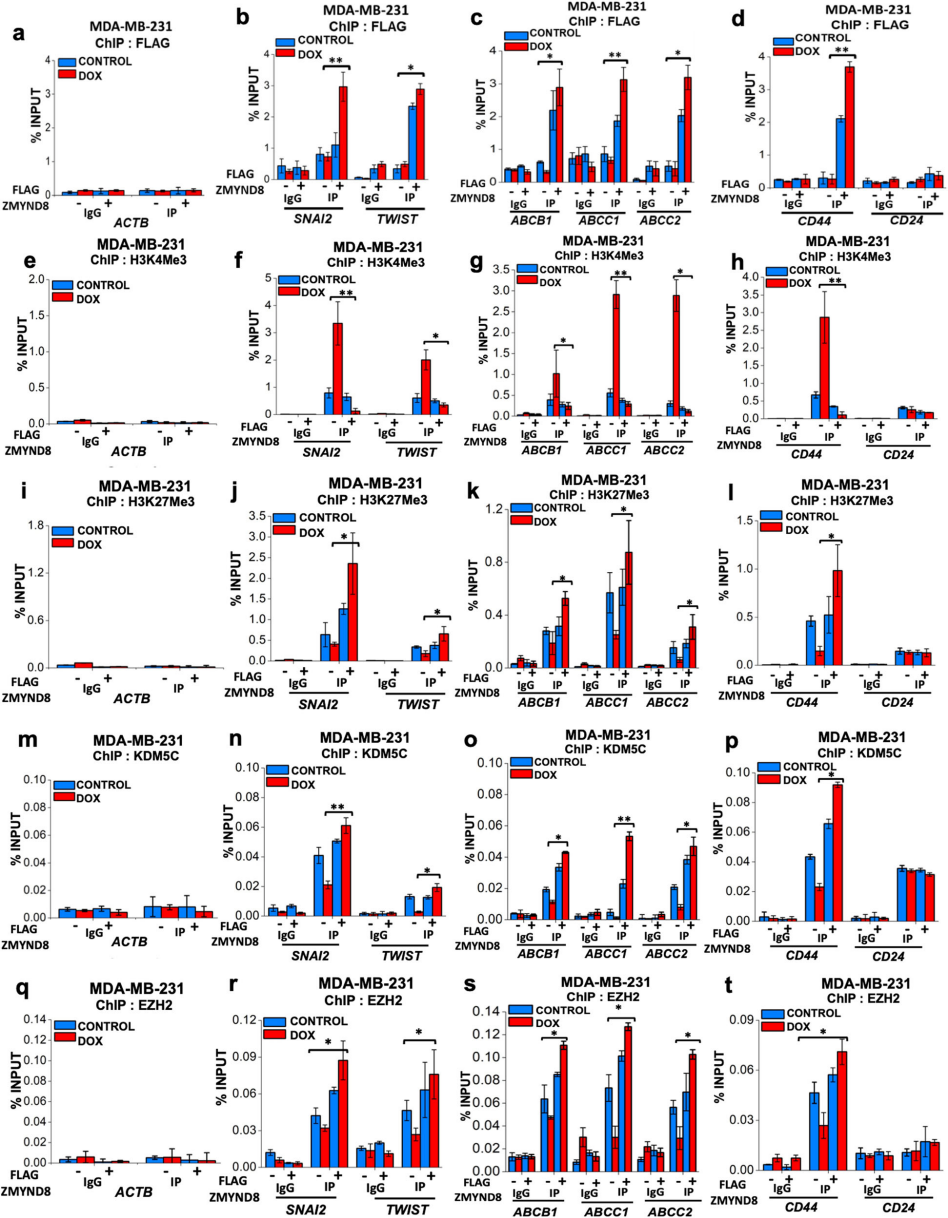
ZMYND8 acts as a chemo-sensitizer by modulating poised tumor-promoting genes. **a**–**t** Bar plots for qPCR enrichment of FLAG-ZMYND8 (**a–d**), H3K4Me3 (**e–h**), H3K27Me3 (**i–l**), KDM5C (**m–p**), or EZH2 (**q–t**) ChIP in MDA-MB-231 cells on stemness, drug resistance, and EMT genes upon FLAG-ZMYND8 overexpression followed by doxorubicin treatment (0.6 µM for 48 h). *ACTB* was used as a negative control. Error bars indicate standard deviation (s.d.); *n* = 3 technical replicates of a representative experiment (out of three experiments). *P* values were calculated using one-way ANOVA. *P < 0.05 and **P < 0.01.

Previous reports showed that the initiation of EMT and stemness by extracellular signal takes place through activation of poised state promoter of ZEB1^43^. This led us to hypothesize that ZMYND8 overexpression represses the tumor-promoting target gene expression through the alteration of poised epigenetic states of their promoters. We observed that ZMYND8 overexpression significantly increased the recruitment of ZMYND8 to EMT, drug resistance, and stemness-related gene promoters as compared to doxorubicin treatment alone in MDA-MB-231 cells (Fig. 7a–d). Notably, a decrease in H3K4Me3 (Fig. 7e–h) and increase in H3K27Me3 (Fig. 7i–l) occupancy was observed at the promoters of these target genes upon ZMYND8 overexpression followed by doxorubicin treatment. In the next step, we sought to delineate the epigenetic regulators that are involved in the above-mentioned gene expression changes. The transcription activation mark, H3K4Me3, can be removed by a few histone modifiers, including those of the KDM or JARID family^44–46^, which eventually leads to transcriptional repression. Among these, KDM5C (lysine demethylase 5C) has been reported to have a significant role in transcriptional regulation of cancer cells in concert with ZMYND8^24,26^. Again, H3K27Me3 is mediated by the specific histone methyltransferase EZH2 (Enhancer of zeste homolog 2), which causes transcriptional repression^47,48^. In order to decipher the mechanism of altered occupancy of H3K4Me3 and H3K27Me3 upon ZMYND8 overexpression followed by doxorubicin treatment, we investigated the recruitment of these epigenetic modulators under similar conditions. Concomitantly, an increase in KDM5C (Fig. 7m–p) and EZH2 (Fig. 7q–t) occupancy at the EMT, drug resistance, and stemness-related gene promoters were observed in MDA-MB-231 cells. Therefore, our results showed that ZMYND8 regulates the poised epigenetic state of these tumor-promoting genes by modulating their respective epigenetic regulators, KDM5C and EZH2.

### ZMYND8 forms transcriptional repressor complex with KDM5C and EZH2 and downregulates the expression of tumor-promoting genes

**Fig. 8:**
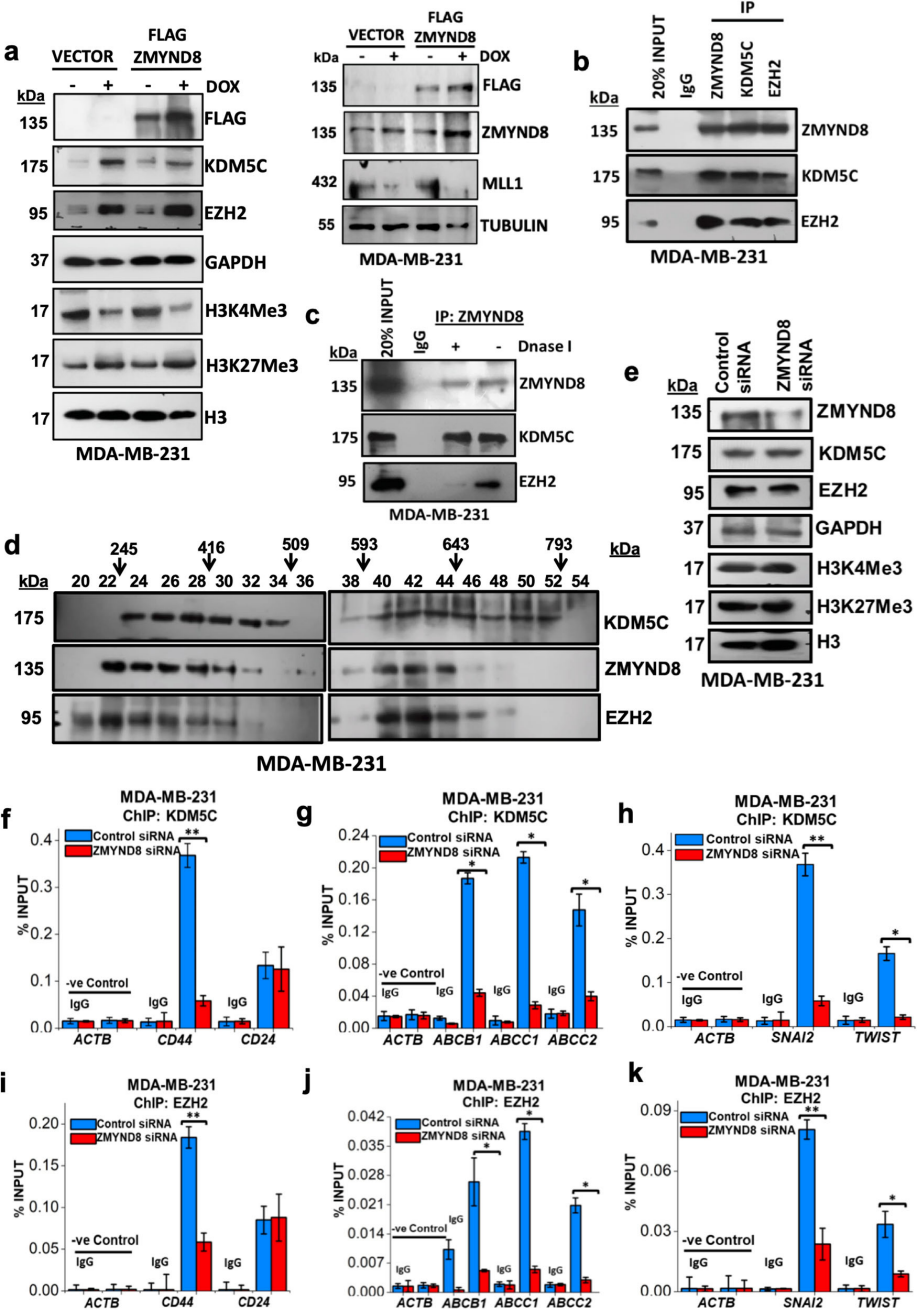
ZMYND8 regulates poised tumor-promoting genes through KDM5C and EZH2. **a** Immunoblots depicting the expression of KDM5C, EZH2, H3K4Me3, H3K27Me3, ZMYND8, and MLL1 upon doxorubicin treatment (0.6 μM for 48 h) in ZMYND8-overexpressed MDA-MB-231 cells. H3, TUBULIN and GAPDH were used as control. **b** Co-immunoprecipitation of ZMYND8, KDM5C, EZH2, or IgG (negative control) from MDA-MB-231 cells was analyzed by immunoblotting. **c** DNase I co-immunoprecipitation of ZMYND8, KDM5C, EZH2, or IgG (negative control) from MDA-MB-231 cells was analyzed by immunoblotting. **d** Immunoblots with ZMYND8, KDM5C, and EZH2 of sucrose-gradient fractions of lysate from MDA-MB-231 cells. **e** Immunoblots depicting the expression of KDM5C, EZH2, H3K4Me3, H3K27Me3 upon ZMYND8 knock down in MDA-MB-231 cells. H3 and GAPDH were used as control. **f–k** Bar plot for qPCR enrichment of KDM5C (**f–h**) or EZH2 (**i–k**) on stemness, drug resistance, and EMT genes in MDA-MB-231 cells expressing Control siRNA or ZMYND8 siRNA. Error bars indicate standard deviation (s.d.); *n* = 3 technical replicates of a representative experiment (out of three experiments). *P* values were calculated using unpaired Student’s *t*-tests. *P < 0.05 and **P < 0.01.

Next, we wanted to determine whether ZMYND8 regulates H3K4Me3 and H3K27Me3 marks at global levels and also their modifiers in sublethal doxorubicin-treated MDA-MB-231 cells. Interestingly, there was no significant alteration globally in levels of H3K4Me3, H3K27Me3 or their regulators, KDM5C or EZH2, upon ZMYND8 overexpression alone in MDA-MB-231 cells. However, doxorubicin treatment triggered the expression of ZMYND8, or the epigenetic regulators, KDM5C or EZH2, and consequently their cognate epigenetic signatures H3K4Me3 and H3K27Me3 (Fig. 8a). Furthermore, we found an enhanced occupancy of RNA polymerase II S5phospho at the KDM5C and EZH2 promoters upon doxorubicin treatment alone or in combination with ZMYND8 overexpression. However, ZMYND8 overexpression alone leads to a decreased occupancy of RNA polymerase II S5phospho at KDM5C and EZH2 promoters, whereas non-phosphorylated RNA polymerase II remains unaltered. This result clearly indicates that ZMYND8 overexpression has no significant effects on elevated expression of KDM5C or EZH2, which occurs only due to the low dose of doxorubicin treatment (Supplementary Fig. 9a, b). Similarly, we verified whether the doxorubicin-specific effect could also be seen upon 5-FU treatment at a sublethal dose in MDA-MB-231 cells. Interestingly, we found that similar to doxorubicin, a sublethal dose of 5-FU alone or in combination with ZMYND8 altered the levels of H3K4Me3, H3K27Me3, KDM5C, and EZH2 in MDA-MB-231 cells (Supplementary Fig. 9c).

A recent report suggests that KDM5C forms a corepressor complex with ZMYND8, and mediates the repression of metastasis promoting genes in breast cancer^26^. In our study, we also observed a strong association of KDM5C with ZMYND8 in MDA-MB-231 cells (Fig. 8b). Additionally, we found that the ZMYND8 and KDM5C complex immunoprecipitates EZH2 from MDA-MB-231 cells (Fig. 8b). Although ZMYND8 failed to immunoprecipitate EZH2 after DNase I digestion, its association with KDM5C remained unaltered (Fig. 8c). This indicates that the association of ZMYND8 with EZH2 was possibly a chromatin-template-dependent phenomenon. However, ZMYND8 showed an enhanced association with KDM5C and EZH2 upon doxorubicin treatment at a sublethal dose in MDA-MB-231 cells (Supplementary Fig. 9d). Our newly identified co-repressor complex comprising ZMYND8, KDM5C, and EZH2 was further validated by sucrose-gradient fractionation assay (Fig. 8d). Although upon loss of ZMYND8, the global levels of these epigenetic modulators KDM5C and EZH2 and their corresponding signatures H3K4Me3 and H3K27Me3 remain unaltered (Fig. 8e), a diminished occupancy of these corepressors from the EMT, drug resistance, and stemness-related gene promoters (Fig. 8f–k) was observed. Interestingly, we found the removal of H3K27Me3 was observed upon KDM5C knockdown; conversely, an enhanced enrichment of H3K4Me3 upon loss of EZH2, from these target gene promoters (Supplementary Fig. 10a, b). KDM5C and EZH2 knockdown led to an increased H3K4Me3 and decreased H3K27Me3 occupancy respectively from these promoters, as expected (Supplementary Fig. 10c, d). This indicates that both EZH2 and KDM5C are required for maintaining the genes in a repressed state. However, the global levels of H3K27Me3 or H3K4Me3 remain unaltered upon KDM5C and EZH2 knockdown, respectively (Supplementary Fig. 10e, f). We also checked for ZMYND8 dependency in the recruitment of methyltransferase MLL1 or demethylases KDM6A and KDM6B for their respective histone marks H3K4Me3 or H3K27Me3 on these target gene promoters. ZMYND8 overexpression has no significant changes in their recruitment to the target gene promoters was observed (Supplementary Fig. 10g–o). Therefore, our results indicate that ZMYND8 in association with the corepressor complex (KDM5C and EZH2) transcriptionally represses the poised tumor-promoting genes by removing H3K4Me3 and reinstating H3K27Me3 levels.

## Discussion

ZMYND8 is a putative chromatin reader and has a tumor-suppressive role by suppressing the metastasis-linked genes and reinstating the epithelial state of the cells^24,25^. Previously, we have shown that in luminal breast cancer cells, ZMYND8 regulates the migratory potential and suppresses EMT through its chromatin reader function^25^. Other reports suggest that it suppresses the metastasis-linked genes through chromatin recognition function in prostate cancer^24^. Recently an oncogenic function of ZMYND8 has been reported where acetylated ZMYND8 activates HIF1α transcriptionally, and promotes angiogenesis^49^. Again, ZMYND8 inhibits cancer cell proliferation via transcription inhibition in response to chemotherapeutic drug all-*trans* retinoic acid^50^. In the present study, we demonstrate a novel anti-chemo-resistance role of ZMYND8, where through its repressive function it re-sensitizes breast cancer cells to chemotherapy.

Earlier studies have revealed that a low dose of chemotherapeutic drug doxorubicin reduces cell death, but increases drug resistance, stemness, and migration properties^51,52^. Both our in vitro and in vivo findings reveal that such treatment-induced “acquired resistant” phenotype leads to enhanced tumorigenic potential. Interestingly, ZMYND8 not only reversed the doxorubicin-induced drug-resistant phenotypes, it further reinstated chemo-responsive state in these metastatic cells. Of note, we also observed that ZMYND8 alone could regulate the expression of DNA-damage-related genes (Supplementary Fig. 5d), which is in accordance with the previous reports^53^. But this effect had no bearing on the doxorubicin-induced levels, suggesting ZMYND8 induction is necessary but not sufficient to control DNA-damage response. Also, previous study has reported that a cytosolic fraction of ZMYND8 is sequestered by Drebrin^54^. We also observed that although ZMYND8 is upregulated upon doxorubicin treatment, yet ZMYND8 gets removed from the EMT, MDR, and CSC gene promoters. This could be possibly in part due to induction of NFAT^55^ and AREB6 (ZEB1)^56^ which targets pro-survival genes upon doxorubicin treatment, which could play a critical role in negating the effect of endogenous ZMYND8. Further, a possible mode could also be through a cytosolic translocation of a subpopulation of ZMYND8 upon dox treatment where it performs some additional function. As observed previously, acetylated ZMYND8 could also play a role in regulating its tumorigenic potential in terms of recruitment/removal onto/from its target promoters. These are avenues of future research which needs further investigation. Taken together, these findings indicate potent chemo-sensitizing potential of ZMYND8, which is instrumental in improving the effective dose of the chemotherapeutic drug, thereby curtailing the extent of systemic toxicity and undesirable off-target effects.

In the present study, we delineate a novel epigenetic mechanism of chemo-resistance in breast cancer cells, which is pre-disposed to specific tumor-promoting genes in cancer cells for prompt transcription activation of “poised” chromatin states. Tumor-promoting genes positively mediating the acquisition of chemo-resistance are maintained in a poised state primed to be activated by chemotherapy. A poised/bivalent promoter is a distinguishing characteristic of developmental genes, which were originally identified in mouse embryonic stem cells. The combination of both activating H3K4Me3 and repressive H3K27Me3 marks in bivalent promoters maintain genes in a poised state; bivalent promoters are pre-loaded with paused RNA Polymerase II that keeps the gene “poised” for quick firing and transcriptional activation^57^. A few studies have reported bivalent promoters in cancer cells^58–60^, and this bivalency has been correlated with gene silencing through hypermethylation^61^. However, the salient finding of our work is how poised chromatin state is repressed by ZMYND8, which otherwise gets activated by chemotherapy, leading to chemo-resistance.

ZMYND8 recognizes H3K36Me2, H4K16Ac, H3K4Me1, and H3K14Ac, exhibits both transcriptional activation and repression^23,24^. Previous reports have elucidated that the activating mark, H3K4Me3^62^, and repressive mark, H3K27Me3^47,48^, are altered by epigenetic modifiers to regulate transcription. ZMYND8 exhibits its repressor function by associating with KDM5C and JARID1D, and we identified its association with EZH2 in a chromatin dependent manner. Our findings showed that ZMYND8 is instrumental in recruiting corepressors, KDM5C, and EZH2 onto their target promotors to regulate tumor-genes in breast cancer. Collectively, our work identifies ZMYND8 as an epigenetic therapeutic tool that can be used in combination with chemotherapy for combating breast cancer. There is an ample scope to improve the outcome of chemotherapy in breast cancer, in particular, the frequent occurrence of “acquired drug resistance” post-chemotherapy. Keeping in mind, the undesirable systemic toxicity of chemotherapeutic drugs in patients, we have shown how ZMYND8 in combination with sublethal dose of the chemotherapeutic drug can modulate the epigenome of the breast cancer cells, reversing the acquisition of chemo-resistance. Whether ZMYND8 can exert chemo-sensitization effects in a clinical scenario and in the presence of different chemotherapeutic regimens needs further investigation.

## Supplementary information

ZMYND8 loss promotes stemness, drug resistance and EMT

Resistant property is acquired by cancer cells by low dosage of chemotherapeutic drugs.

Genome wide changes upon doxorubicin treatment in ZMYND8-overexpressed cells

ZMYND8 / doxorubicin-regulated genes highlighted in red star in cancer pathways as analyzed by DAVID tool.

Canonical pathways effected by ZMYND8- and/or doxorubicin-downregulated genes.

ZMYND8 and/or doxorubicin regulated genes predict clinical outcomes.

ZMYND8 induce chemo sensitization by various chemotherapeutic drugs.

In vivo and in vitro validation of chemo sensitization by ZMYND8.

Doxorubicin and 5FU elicits KDM5C and EZH2 expression, with an enhanced association with ZMYND8.

ZMYND8 associates with KDM5C and EZH2 to maintain the poised epigenetic state at tumor promoting genes

Supplementary Figure Legends

List of antibodies

List of primers used for qRT-PCR and ChIP qPCR

Accession id and expression values of ZMYND8 in Reference tumor, Patient with Miller-Payne index 4 & 5, Patients with Miller-Payne index 1

Accession id and expression values of ZMYND8 in TNBC patients with recurrent and non-recurrent tumor
