# Supplementary figures and images for: Suppression of poised oncogenes by ZMYND8 promotes chemo-sensitization
Source: Cell Death Dis. 2020 Dec 15;11(12):1073. doi: 10.1038/s41419-020-03129-x (PMC7738522; doi:10.1038/s41419-020-03129-x)

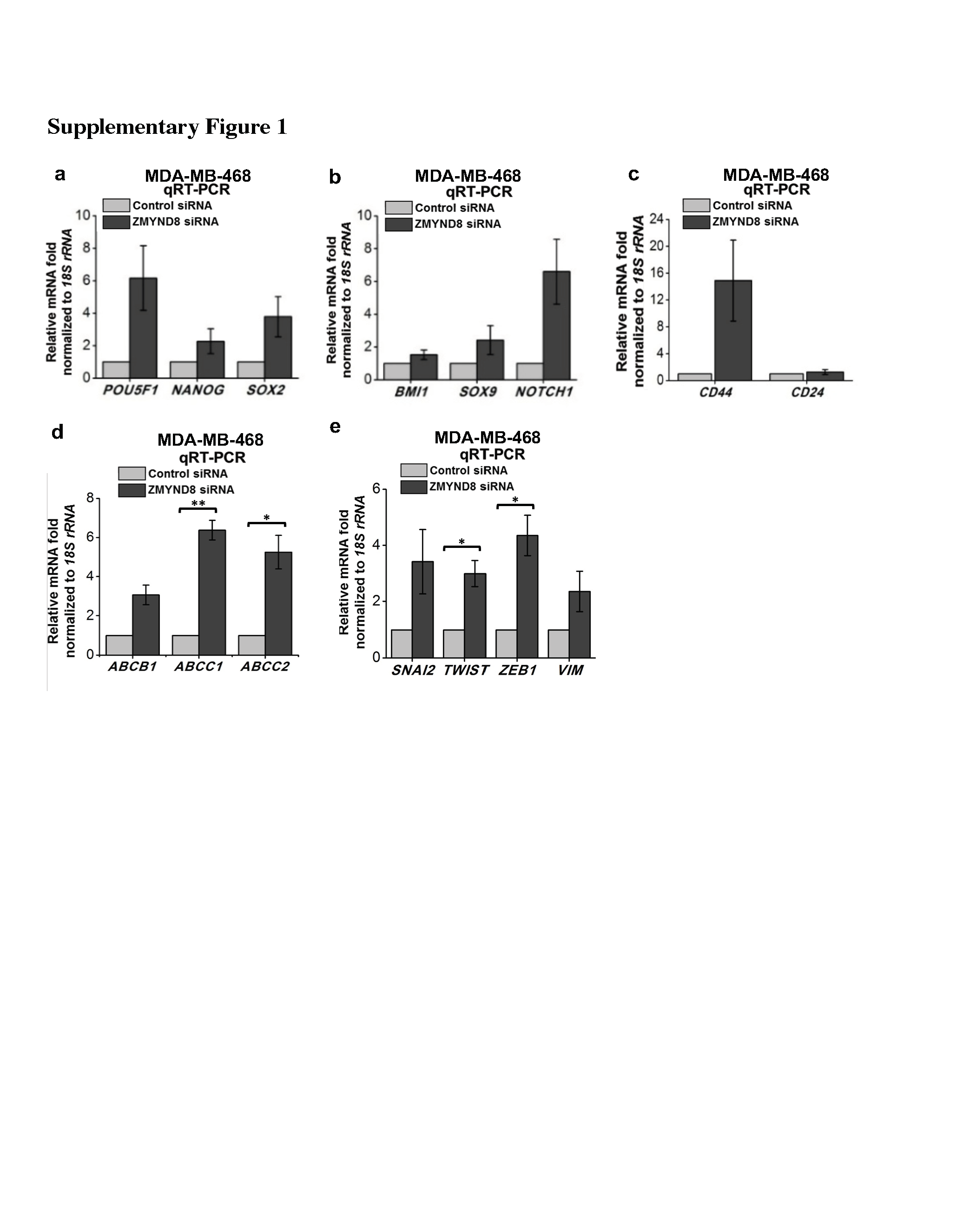

Supplement: Supplementary file 1 — ZMYND8 loss promotes stemness, drug resistance and EMT [file 41419_2020_3129_MOESM1_ESM.tif]

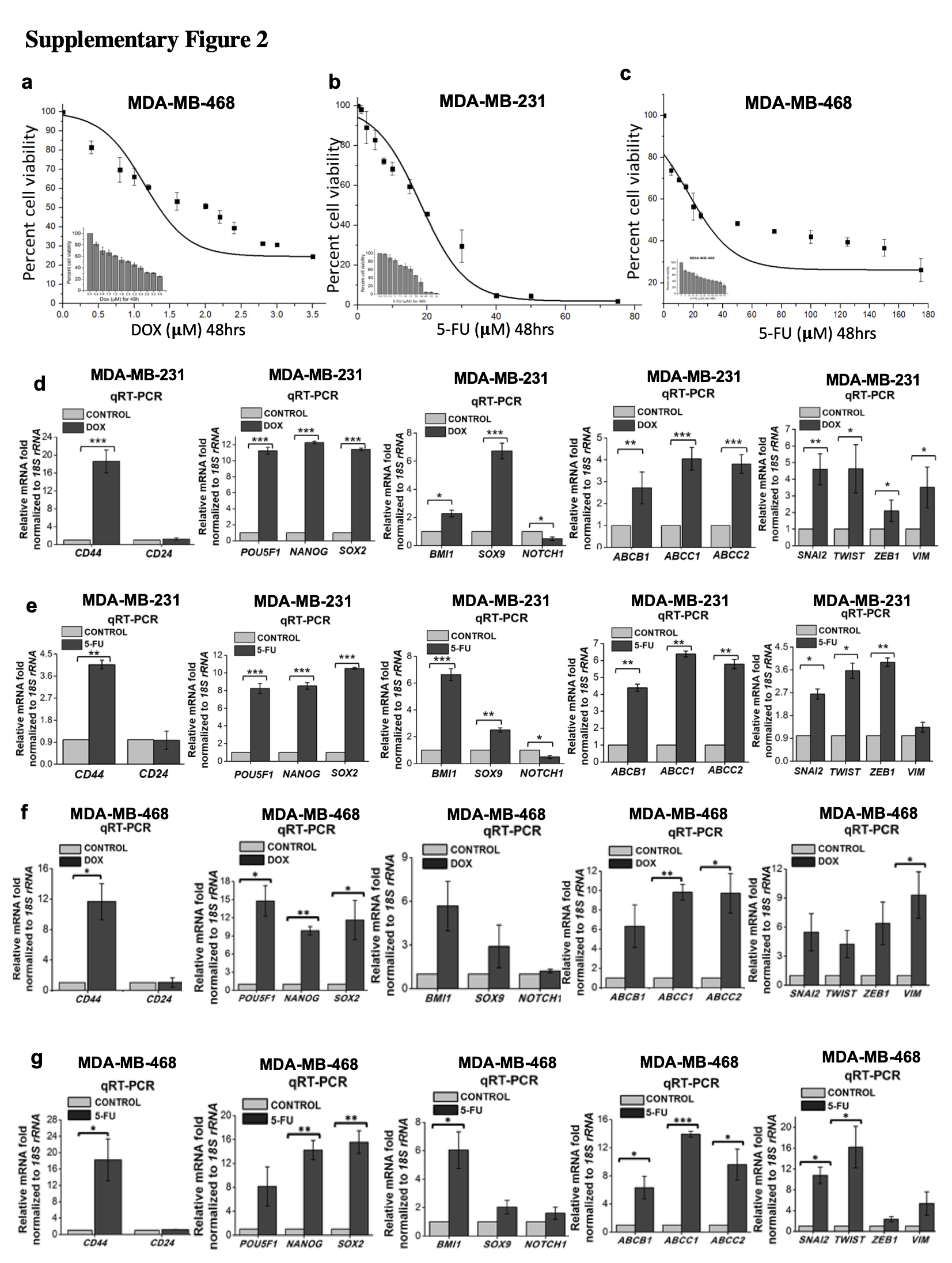

Supplement: Supplementary file 2 — Resistant property is acquired by cancer cells by low dosage of chemotherapeutic drugs. [file 41419_2020_3129_MOESM2_ESM.tif]

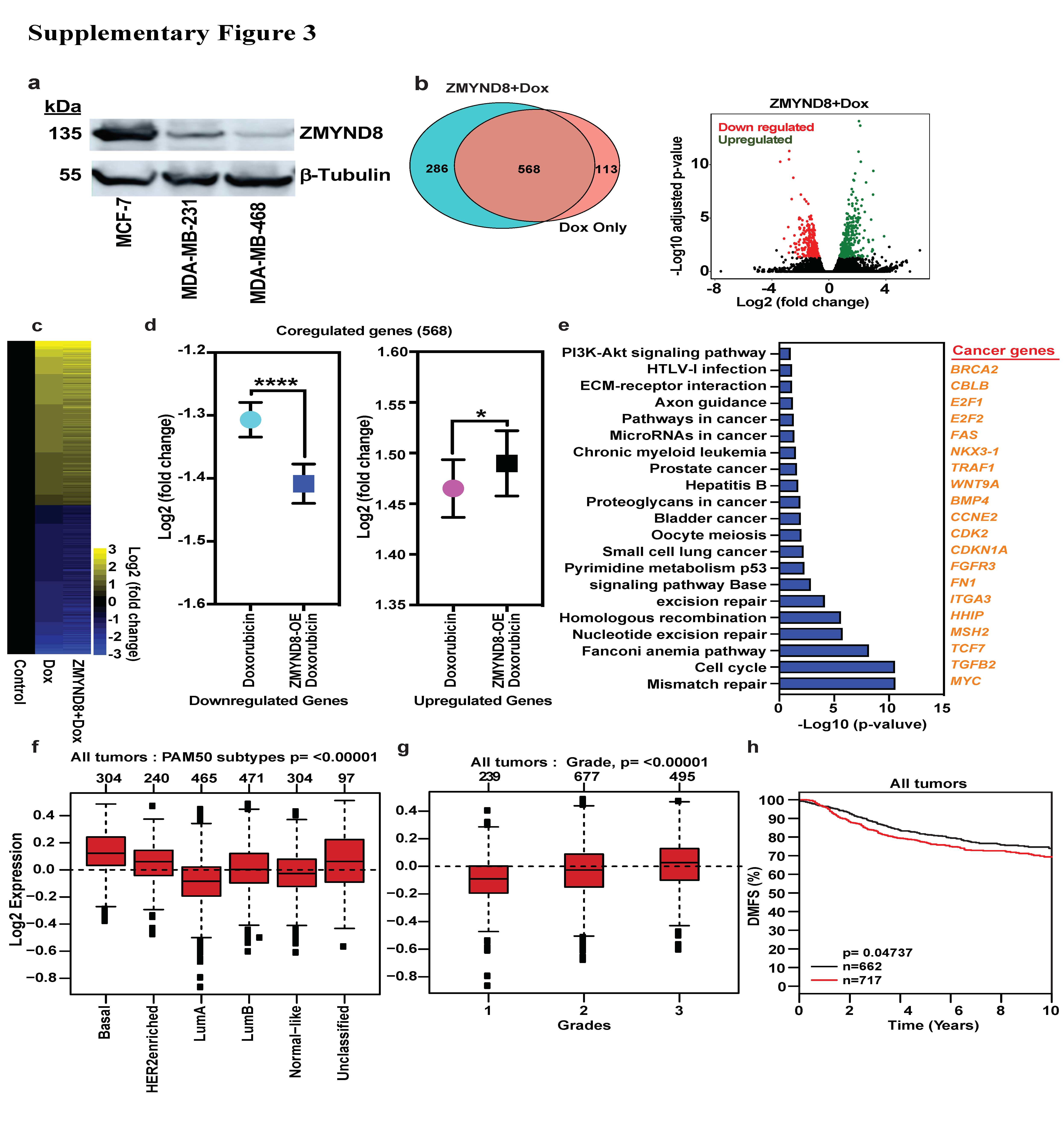

Supplement: Supplementary file 3 — Genome wide changes upon doxorubicin treatment in ZMYND8-overexpressed cells [file 41419_2020_3129_MOESM3_ESM.tif]

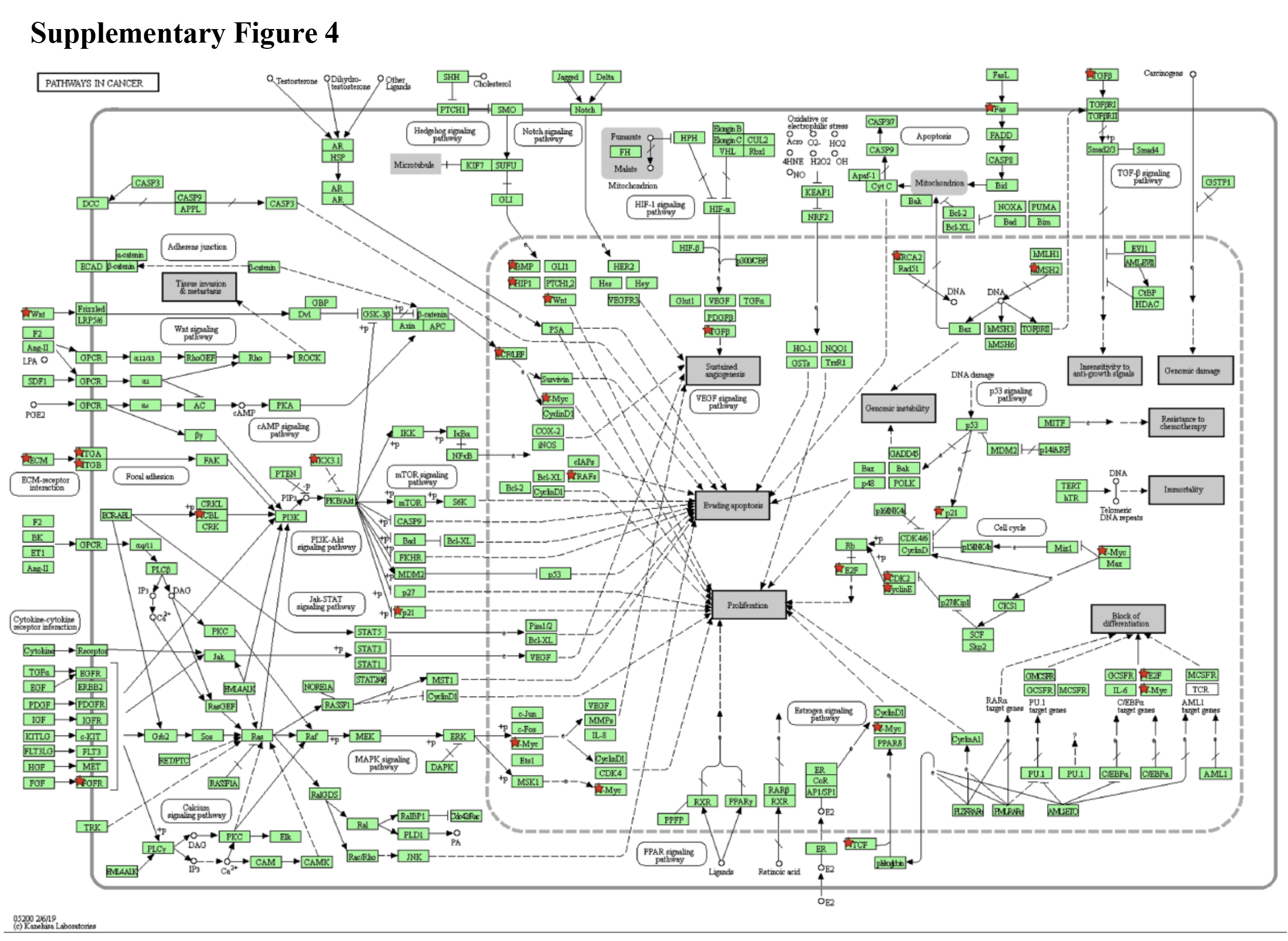

Supplement: Supplementary file 4 — ZMYND8 / doxorubicin-regulated genes highlighted in red star in cancer pathways as analyzed by DAVID tool. [file 41419_2020_3129_MOESM4_ESM.tif]

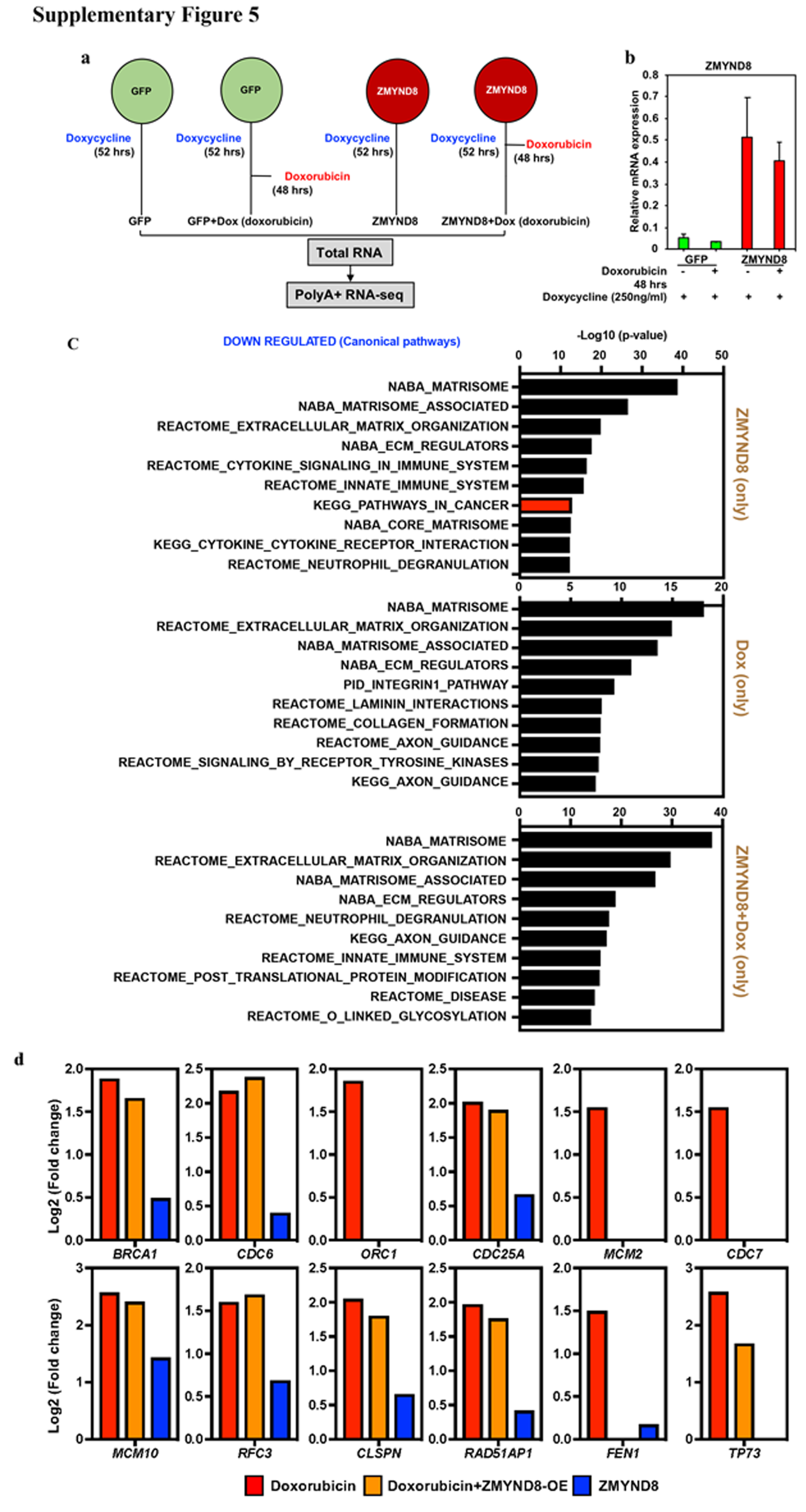

Supplement: Supplementary file 5 — Canonical pathways effected by ZMYND8- and/or doxorubicin-downregulated genes. [file 41419_2020_3129_MOESM5_ESM.tif]

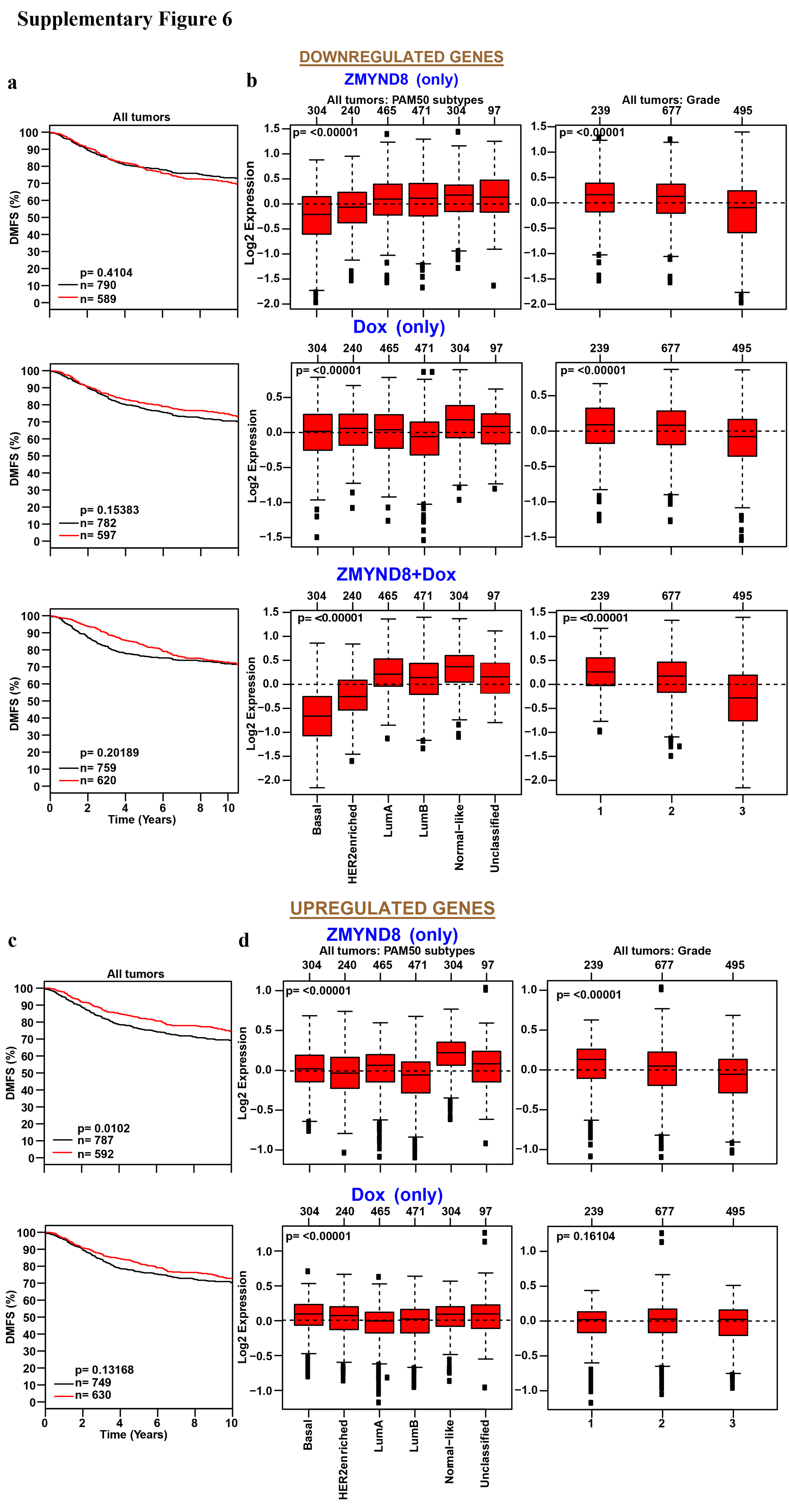

Supplement: Supplementary file 6 — ZMYND8 and/or doxorubicin regulated genes predict clinical outcomes. [file 41419_2020_3129_MOESM6_ESM.tif]

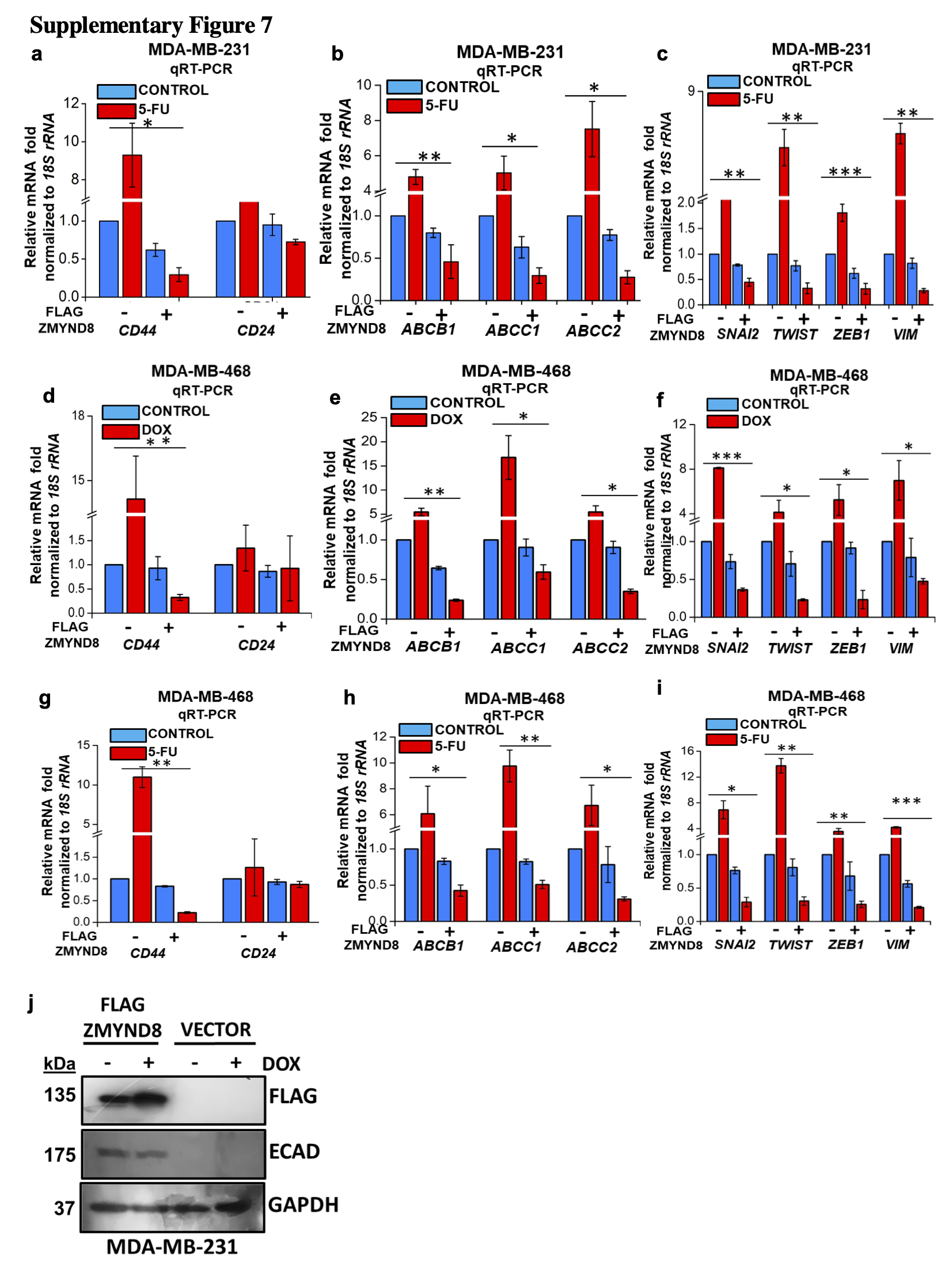

Supplement: Supplementary file 7 — ZMYND8 induce chemo sensitization by various chemotherapeutic drugs. [file 41419_2020_3129_MOESM7_ESM.tif]

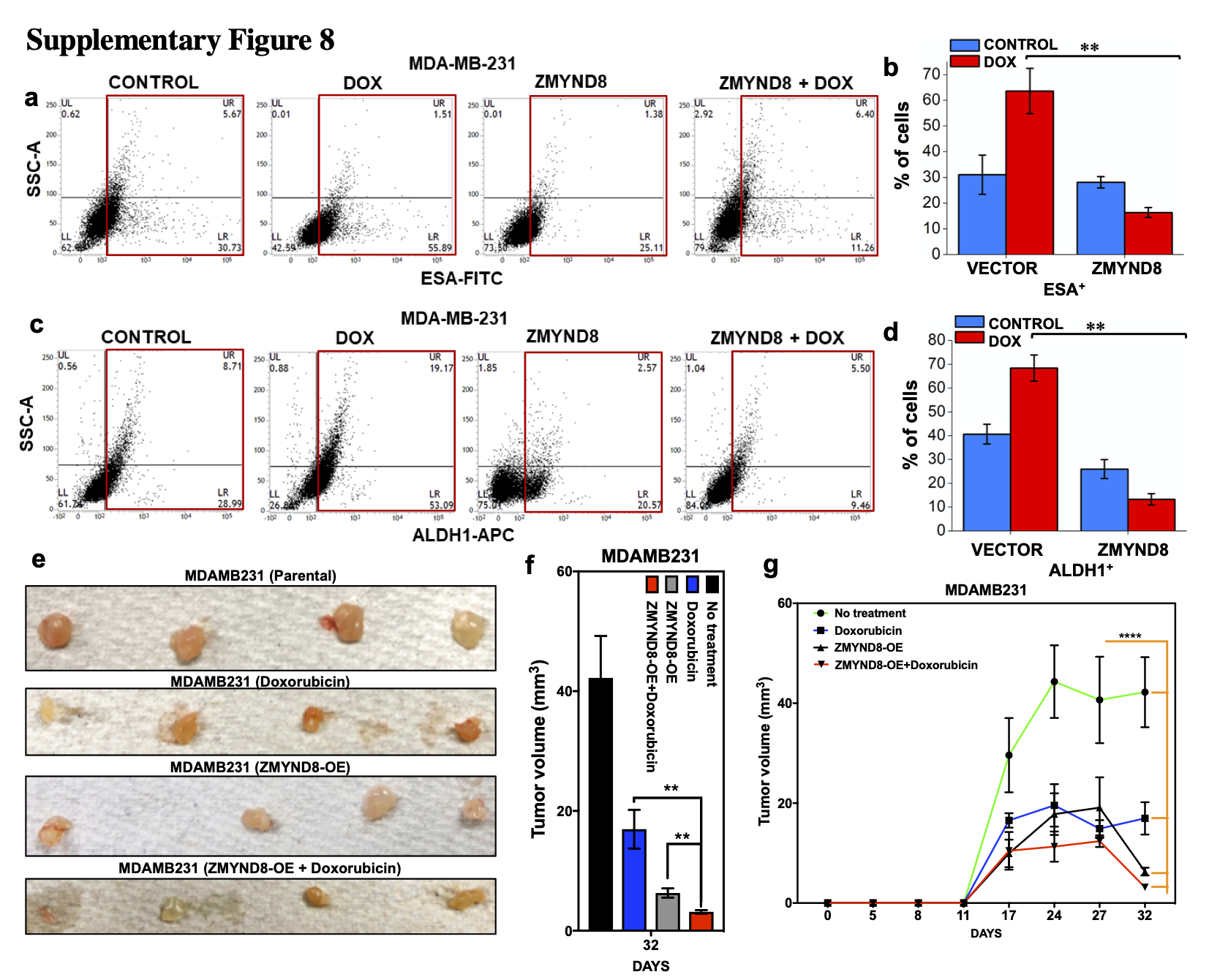

Supplement: Supplementary file 8 — In vivo and in vitro validation of chemo sensitization by ZMYND8. [file 41419_2020_3129_MOESM8_ESM.tif]

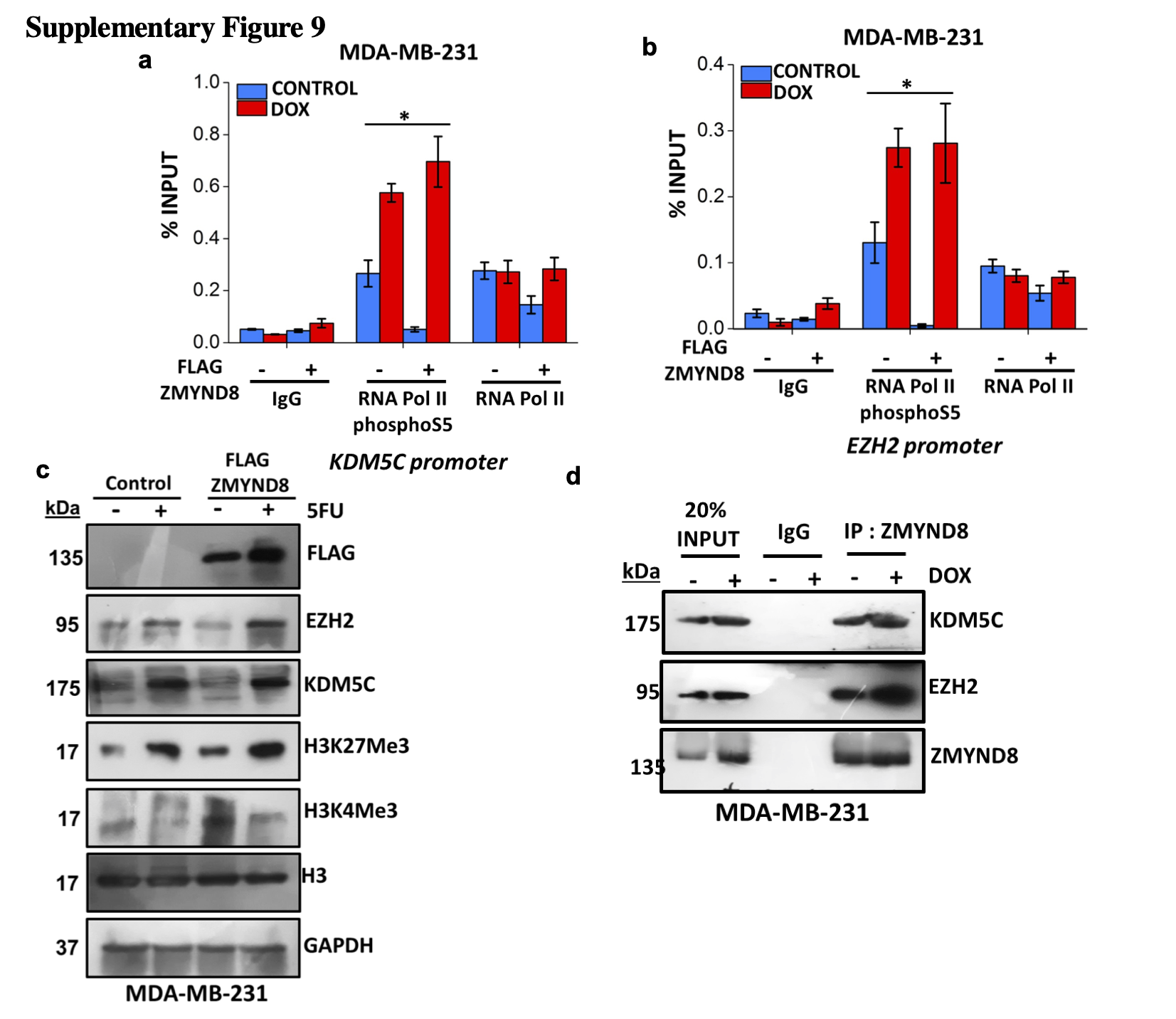

Supplement: Supplementary file 9 — Doxorubicin and 5FU elicits KDM5C and EZH2 expression, with an enhanced association with ZMYND8. [file 41419_2020_3129_MOESM9_ESM.tif]

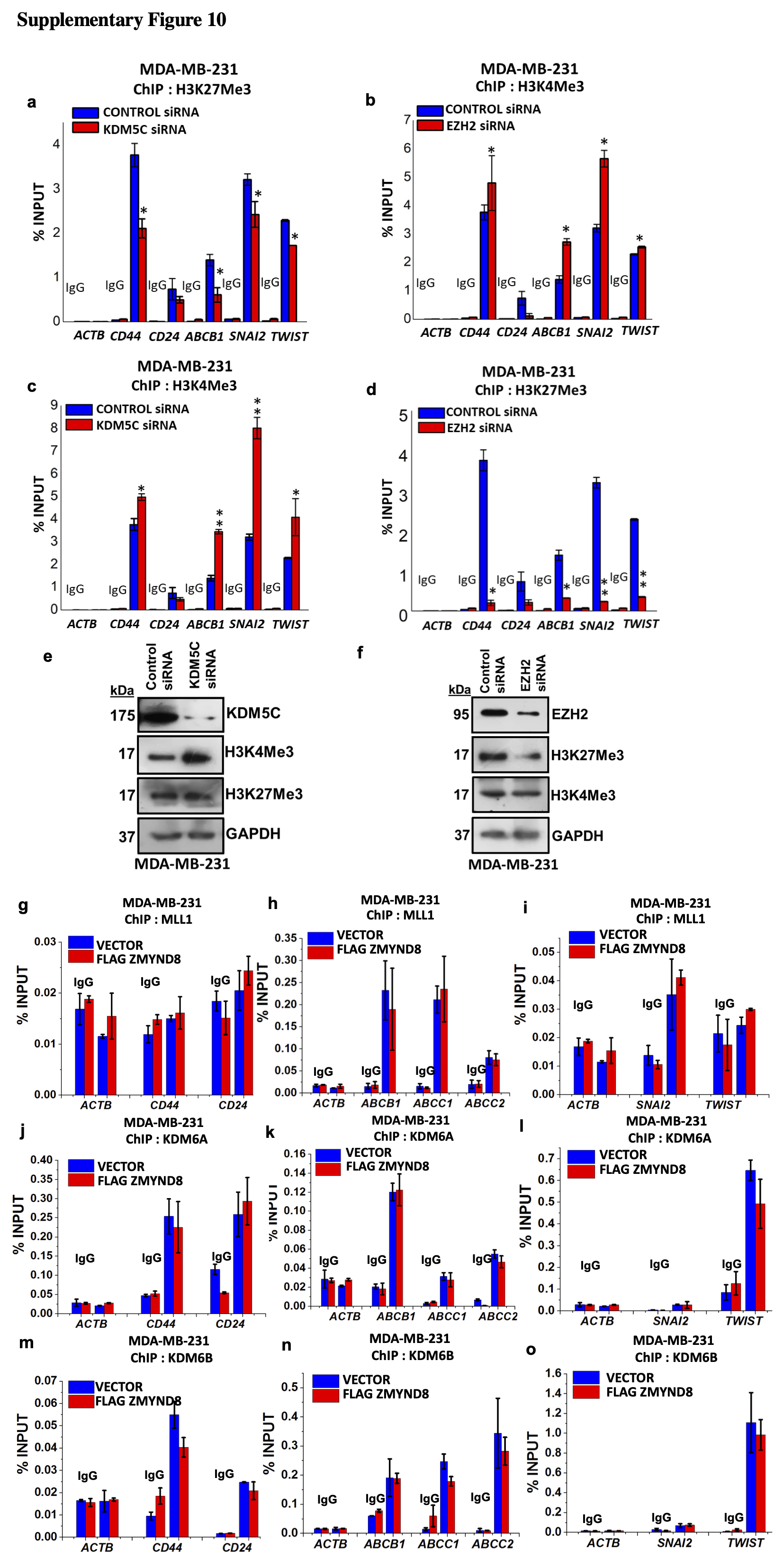

Supplement: Supplementary file 10 — ZMYND8 associates with KDM5C and EZH2 to maintain the poised epigenetic state at tumor promoting genes [file 41419_2020_3129_MOESM10_ESM.tif]
