# Supplementary material for: Suppression of poised oncogenes by ZMYND8 promotes chemo-sensitization
Source: Cell Death Dis. 2020 Dec 15;11(12):1073. doi: 10.1038/s41419-020-03129-x (PMC7738522; doi:10.1038/s41419-020-03129-x)
Supplement: Supplementary file 11 — Supplementary Figure Legends [file 41419_2020_3129_MOESM11_ESM.docx]

**Supplementary Figure Legends**

**Supplementary Figure 1: ZMYND8 loss promotes stemness, drug resistance and EMT. a-e** qRT-PCR analysis showing the expression of pluripotency / stemness related **(a-c)**, drug resistance **(d)** and EMT **(e)** genes upon ZMYND8 knockdown via siRNA in MDA-MB-468 cells. In all panels error bars indicate standard deviation (s.d.); n=3, a representative with technical replicates (out of three experiments). *P*-values were calculated using unpaired Student’s *t*-tests. **P*<0.05; ***P*<0.01; ****P*<0.001.

**Supplementary Figure 2: Resistant property is acquired by cancer cells by low dosage of chemotherapeutic drugs. a-c** ~~MDA-MB-231~~ MDA-MB-468 cells were treated with increasing doses of ~~5-Flurouracil~~ doxorubicin **(a)** for 48hrs and ~~MDA-MB-468~~ MDA-MB-231 **(b)** or MDA-MB-468 **(c)** cells were treated with increasing doses of ~~doxorubicin or~~ 5-Flurouracil for 48hrs. Cell viability was measured by MTT assay. **d-g** qRT-PCR analysis showing expression of pluripotency / stemness related, drug resistance and EMT genes upon 0.6µM doxorubicin **(d)** or 10µM 5-Fluorouracil treatment for 48hrs in MDA-MB-231 cells **(e)** or 1.0µM doxorubicin **(f)** or 20µM 5-Fluorouracil **(g)** treatment for 48hrs in in MDA-MB-468 cells.

**Supplementary Figure 3: Genome wide changes upon doxorubicin treatment in ZMYND8 overexpressed cells. a** Immunoblot analysis of ZMYND8 in MCF-7, MDA-MB-231 and MDA-MB-468 cells. **b** Venn diagram depicting common and specific genes regulated by ZMYND8, ZMYND8 doxorubicin (dox) and dox. Volcano plot showing ~~upon~~ down-regulated genes upon doxorubicin treated ZMYND8 overexpressed MDA-MB-231 cells. **c** Heat map of differentially expressed genes (log2 fold change ≥ 1, and P-value ≤ 0.05) upon doxorubicin (dox) treatment in ZMYND8 overexpressed MDA-MB-231 cells. **d** Cumulative fold change of all the significantly-coregulated genes. Each symbol represents the mean ± SEM. Regulation in ZMYND8 overexpressed condition is significantly different than dox alone (Student’s paired two-tailed t test; p value < 0.05). **e** Gene Ontology (Biological process) categories showing highest enrichment in a list of DEGs obtained after RNA-Seq analysis of ZMYND8 overexpressed, and sub-lethal dose of doxorubicin treated MDA-MB-231 cells. GO terms are indicated in the Y-axis. P-value at X-axis indicates the significance level of each pathway as obtained from DAVID tool. The list of key cancer related genes has been shown. **f** The ZMYND8/doxorubicin-coregulated genes expressed in tumor samples of the same basal breast cancer molecular subtype. Observed differences are significant as determined by an ANOVA comparison of the means (P-value < 0.00001). **g** The ZMYND8/doxorubicin-coregulated genes in MDA-MB-231 cells are more highly expressed in higher grade tumor samples compared to the lower grades. Observed differences are significant as determined by an ANOVA comparison of the means (P-value < 0.00001). **h** Kaplan-Meier survival analyses of patients expressing high levels of ZMYND8/doxorubicin coregulated gene mRNAs (red line) exhibit a poorer outcome compared to patients expressing low levels of coregulated gene mRNAs (black line). The breast cancer outcome linked gene expression data were accessed and graphed using the Gene Expression-Based Outcome for Breast Cancer Online (GOBO) tool.

**Supplementary Figure 4: ZMYND8 / doxorubicin-regulated genes highlighted in red star in cancer pathways as analyzed by DAVID tool.**

**Supplementary figure 5: Canonical pathways effected by ZMYND8- and/or doxorubicin-downregulated genes. a** Flow chart depicting polyA+ RNA-seq analysis upon ZMYND8 overexpression and/or doxorubicin treatment in doxycycline inducible GFP- or ZMYND8-overexpression MDA-MB-231 cell lines. **b** The expression of ZMYND8 was monitored by qRT-PCR analysis in doxycycline inducible GFP or ZMYND8 overexpression MDA-MB-231 cell lines. Each bar represents the mean + SEM, n=2. **c** Gene set enrichment analysis (GSEA) showing canonical pathways enriched from a list of significantly downregulated genes obtained after RNA-Seq analysis upon ZMYND8 overexpression and/or doxorubicin treatment in MDA-MB-231 cells. Canonical pathways terms are indicated in the Y-axis. P-value at X-axis indicates the significance level of each pathway as obtained from ~~DAVID~~ GSEA tool (http://software.broadinstitute.org/gsea/msigdb/annotate.jsp). **d** RNA-seq based expression analysis of key genes upon ZMYND8 overexpression with or without doxorubicin treatment. Represented here is a set of statistically regulated genes obtained from RNA-seq data performed using two biological replicates in respective treatment conditions. Fold change was calculated over normalized read count of candidate genes from GFP expression (control) condition.

**Supplementary figure: 6. ZMYND8 and/or doxorubicin regulated genes predict clinical outcomes. a** Kaplan-Meier survival analyses of patients expressing high levels of downregulated gene mRNAs exhibit no outcome. **b** The downregulated genes ~~expressed in patient tumor samples of the less aggressive breast cancer molecular subtype. These genes~~ in MDA-MB-231 cells are more highly expressed in normal-like patient tumor samples compared to the other tumor types. However, these genes had no effect on patient outcome **(a)**. Observed differences are significant as determined by an ANOVA comparison of the means (P-value < 0.00001). **c** Kaplan-Meier survival analyses of patients expressing high levels of ZMYND8-upregulated gene mRNAs (red line) exhibit a favourable outcome, compared doxorubicin induced gene mRNAs. **d** The ZMYND8 induced genes are expressed in patient tumor samples of the less aggressive breast cancer molecular subtype, and their expression has favourable outcome in breast cancer patients **(c)**. However, dox induced genes preferentially expressed in basal breast cancer subtype. Observed differences are significant except for grades in dox only as determined by an ANOVA comparison of the means (*P*-value < 0.00001). The breast cancer outcome-linked gene expression data were accessed and graphed using the Gene Expression-Based Outcome for Breast Cancer Online (GOBO) tool.

**Supplementary Figure 7: ZMYND8 induce chemo sensitization by various chemotherapeutic drugs. a-i** qRT-PCR analysis showing expression of stemnesss, drug resistance and EMT genes upon ectopic expression of ZMYND8 followed by 10μM 5-Fluorouracil treatment for 48hrs in MDA-MB-231 cells **(a-c)** or 1.0μM doxorubicin **(d-f)** or 20μM 5-Fluorouracil **(g-i)** treatment for 48hrs in in MDA-MB-468 cells. **j** Immunoblot showing expression of E-cadherin upon ZMYND8 overexpressed doxorubicin treated MDA-MB-231 cells. GAPDH was used as loading control.

**Supplementary Figure 8: In vivo and in vitro validation of chemo sensitization by ZMYND8. a-d** FACS analysis showing ESA+ **(a)** and ALDH1+ cells **(c)** from ZMYND8 overexpressed followed 0.6μM doxorubicin treatment MDA-MB-231 cells for 48hrs. The percent cells have been quantified and represented graphically **(b, d). e-g** MDA-MB-231- ZMYND8-copGFP (ZMYND8 over-expressing) cells were injected into the female nude mice subcutaneously. After appearance of tumors, mice were treated with two cycles of doxorubicin at a dose of 8mg/kg body weight on alternate days. Mice were sacrificed after 7 days post-2 cycles of chemotherapy. Left panel shows images of the tumors 7 days post chemotherapy cycle completion (n=3) **(e)**. Graphical representation of tumor volumes shown in the right panel. Error bars denote the standard error of mean derived from four mice per group **(f)**. The significance of differences was assessed in unpaired two tailed Student’s ttests, **p<0.01. Graphical representation of tumor growth rate in the above four sets of nude mice (n=4) **(g)**. The significance of differences between the line curves representing different mice groups was assessed with Two-way ANOVA, ****p<0.0001.

**Supplementary Figure 9: Doxorubicin and 5FU elicits KDM5C and EZH2 expression, with an enhanced association with ZMYND8. a, b** Bar plot for qPCR enrichment of RNA Pol II phospho S5 at KDM5C **(a)** and EZH2 **(b)** gene promoters upon doxorubicin treatment alone or in combination with ZMYND8 overexpression. **c** Immunoblots depicting the expression of KDM5C, EZH2, H3K4Me3, H3K27Me3 upon 5-FU treatment (10μM for 48hrs) in ZMYND8 overexpressed MDA-MB-231 cells. H3 and GAPDH were used as control. **d** Co-Immunoprecipitation of ZMYND8, KDM5C, EZH2 or IgG (negative control) from MDA-MB-231 cells upon doxorubicin treatment (0.6μM for 48hrs) was analysed by immunoblotting. Error bars indicate standard deviation (s.d.); n=3 technical replicates of a representative experiment (out of three experiments).

**Supplementary Figure 10: ZMYND8 associates with KDM5C and EZH2 to maintain the poised epigenetic state at tumor promoting genes. a-d** Bar plots depicting ChIP of H3K27Me3 **(a)** and H3K4Me3 **(b)** upon KDM5C knockdown and ChIP of H3K4Me3 **(c)** and H3K27Me3 **(d)** upon EZH2 knockdown on their target gene promoters in MDA-MB-231 cells. **e, f** Immunoblots showing expression of both H3K4Me3 and H3K27Me3 upon KDM5C knockdown **(e)** and EZH2 knockdown **(f)** in MDA-MB-231 cells. GAPDH was used as loading control. **g-o** Bar plot for qPCR enrichment of MLL1 **(g-i)**, KDM6A **(j-l)** or KDM6B **(m-o)** on stemness, drug resistance and EMT genes in MDA-MB-231 cells expressing VECTOR of FLAG ZMYND8. Error bars indicate standard deviation (s.d.); n=3 technical replicates of a representative experiment (out of three experiments).

**Supplementary Table Legend**

**Supplementary Table S1** : List of antibodies.

**Supplementary Table S2** : List of primers used for qRT-PCR and ChIP qPCR.

**Supplementary Table S3** : (Accession id and expression values of ZMYND8 in Reference

tumor, Patient with Miller-Payne index 4 & 5, Patients with Miller-Payne index 1

[GSE18864]).

**Supplementary Table S4 :** (Accession id and expression values of ZMYND8 in TNBC

patients with recurrent and non-recurrent tumor [GSE43502]).
